# Molecular engineering of CRISPR/Cas12a: from activity enhancement to exponential signal amplification

**DOI:** 10.1039/d6sc02276f

**Published:** 2026-04-27

**Authors:** Qing-Nan Li, Qi-Fan Yang, Wei-Liang Jin, Xiao-Zhe Pang, Wen-Bo Sun, Jia-Xin Wang, Xin-Yue Wang, An-Na Tang, De-Ming Kong, Li-Na Zhu

**Affiliations:** a State Key Laboratory of Medicinal Chemical Biology, Tianjin Key Laboratory of Biosensing and Molecular Recognition, Research Centre for Analytical Sciences, College of Chemistry, Nankai University Tianjin 300071 P. R. China kongdem@nankai.edu.cn; b Department of Chemistry, School of Science, Tianjin University Tianjin 300072 P. R. China linazhu@tju.edu.cn

## Abstract

CRISPR/Cas12a has emerged as a powerful tool for molecular diagnostics, yet its inherent linear signal output and limited amplification efficiency constrain its detection sensitivity. Conventional approaches that rely on coupling with pre-amplification techniques increase operational complexity and introduce potential uncertainties, thereby hindering clinical translation. This review highlights a paradigm shift: moving beyond the canonical “one-target-one-enzyme” model through molecular engineering to intrinsically enhance the catalytic activity of CRISPR/Cas12a and even achieve exponential signal amplification. We systematically summarize key strategies, including crRNA reprogramming, activator strand engineering, reporter probe design, and reaction environment optimization, that collectively enhance Cas12a's kinetics, specificity, and intrinsic signal output. These integrated engineering approaches enable ultrasensitive, pre-amplification-free detection of both nucleic acids and non-nucleic acid targets, paving the way for the next generation of robust and field-deployable point-of-care diagnostics.

## Abstract 2

Schematic illustration of the molecular engineering roadmap for CRISPR/Cas12a-based biosensing, highlighting the progressive transition from activity enhancement to exponential signal amplification.

## Graphical abstract

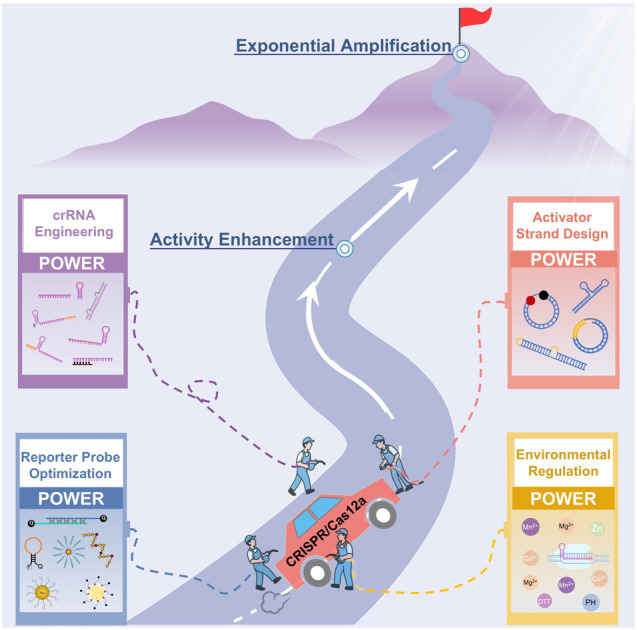

## Introduction

Sensitive, rapid, and specific detection of metal ions, small molecules, and disease-related biomarkers, such as nucleic acids, enzymes, and proteins, is crucial for clinical diagnostics, environmental monitoring, and infectious disease control. Such detection capabilities provide vital information for fundamental biochemical research, drug screening, and early disease diagnosis and treatment.^1–8^ Although polymerase chain reaction (PCR) and isothermal amplification techniques are considered as gold standards due to their high sensitivity and specificity, they still present limitations, including complex operational procedures, reliance on specialized equipment, and potential contamination risks.^9–11^ Consequently, there is a growing interest in developing alternative detection methods that offer rapid, cost-effective, and user-friendly diagnostic solutions.

Against this backdrop, clustered regularly interspaced short palindromic repeats (CRISPR) and CRISPR-associated (Cas) systems have emerged as advanced and highly promising molecular tools. These systems exhibit diverse biological functions and hold great potential for the development of novel biosensors and diagnostic devices.^12–14^ Originally identified as an adaptive immune mechanism in prokaryotes, CRISPR/Cas systems target and cleave foreign genetic elements such as plasmids or phages.^15^ Among various Cas proteins, Cas12a is a type V effector that distinguishes itself with a simple structure and high efficiency, making it a preferred candidate for biomarker detection in biosensing applications.^16,17^

In recent years, the CRISPR/Cas12a system has demonstrated considerable potential in biosensing owing to its unique *trans*-cleavage activity.^18–21^ In this system, Cas12a not only cleaves target double-stranded DNA (dsDNA) *via* its *cis*-cleavage activity but also exhibits nonspecific *trans*-cleavage activity toward single-stranded DNA (ssDNA) upon target recognition.^22,23^ This property has been leveraged to develop biosensors for a wide range of analytes, including nucleic acids,^24–26^ proteins,^27^ small molecules,^28,29^ and ions,^30,31^ highlighting its utility in medical diagnostics, pathogen detection, and environmental monitoring.

Despite these promising advances, CRISPR/Cas12a-based biosensors remain largely confined to the laboratory settings. Two major bottlenecks hinder their practical applications: (1) the inherent limited signal amplification capacity of Cas12a (only 3–17 turnovers per second^23,32^), which is inadequate for detecting low-abundance targets; and (2) heavy reliance on external pre-amplification techniques, such as PCR, recombinase polymerase amplification (RPA), loop mediated isothermal amplification (LAMP), and other exogenous signal amplification strategies (*e.g.*, nanomaterial catalysis or nuclease cascades) to achieve satisfactory sensitivity.^33–35^ These requirements not only increase operational complexity but also raise the risk of cross-contamination, limiting their suitability for point-of-care testing (POCT).

Conventional Cas12a biosensors typically follow a “one-target-one-enzyme” mode. To improve sensitivity, pre-amplification of the target or Cas12a-related components is often necessary. Recently, researchers have turned to engineering the CRISPR/Cas12a system itself to enhance its signal output and reduce dependency on external pre-amplification. These efforts aim to boost catalytic activity and even establish exponential signal amplification mechanisms based on autocatalytic cycles and positive feedback loops. In such systems, target activation triggers the continuous generation of secondary activators, leading to cascaded signal amplification and exponential signal enhancement.

To this end, multiple intrinsic components of the CRISPR/Cas12a system, including crRNA,^36–47^ Cas12a proteins,^48–50^ activator strands^51–63^ and reporter molecules,^26,64–66^ have been systematically engineered. External factors such as ionic conditions^67–71^ have also been optimized to enhance reaction kinetics. Among these, crRNA and activator strand engineering have emerged as particularly powerful strategies, as they directly regulate target binding, conformational activation, cleavage kinetics, and signal propagation behavior. Through terminal extension, site-specific chemical modification, switchable structural programming, and feedback-circuit construction, these molecular designs have enabled Cas12a to evolve from a simple signal transducer into a programmable amplification module. Importantly, such advances suggest that rational molecular engineering can simultaneously improve *trans*-cleavage efficiency, suppress background interference, and establish self-sustaining amplification pathways, thereby opening a route toward ultrasensitive and pre-amplification-free biosensing.

Although several excellent reviews have summarized the general development of CRISPR/Cas12a-based biosensors or their integration with external amplification technologies, this review is conceptually distinct in its specific focus on the intrinsic molecular engineering of the CRISPR/Cas12a system itself. Rather than emphasizing platform assembly or upstream target amplification, we focus on how rational reprogramming of core Cas12a-related components can overcome the inherent linear output of the system and drive the emergence of exponential signal amplification through autocatalytic and positive-feedback mechanisms. This perspective provides a more mechanism-centered framework for understanding how molecular design can fundamentally reshape the performance boundaries of CRISPR/Cas12a biosensing.

**Fig. 1:**
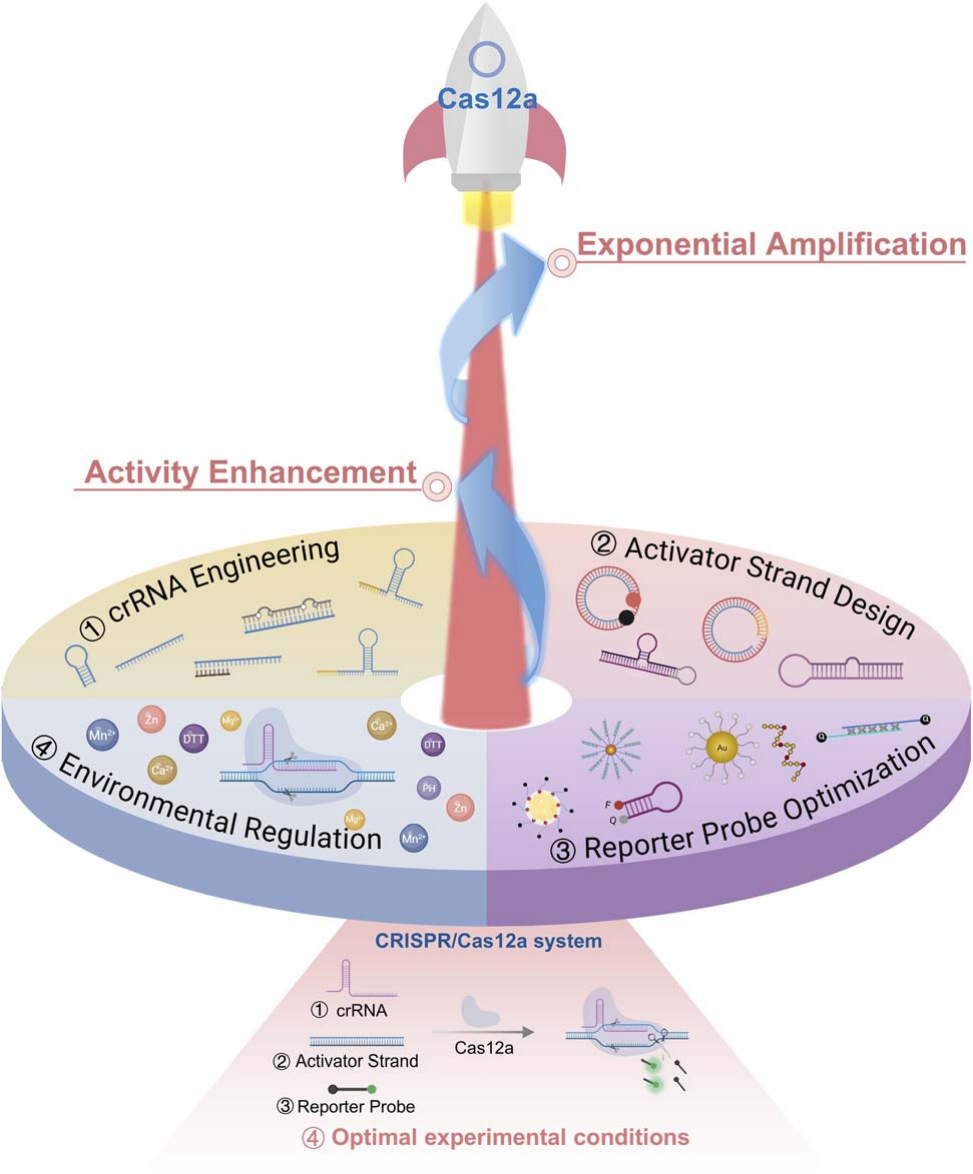
Engineering of core CRISPR/Cas12a components and regulating reaction conditions for activity enhancement and exponential signal amplification.

Based on this framework, we systematically summarize recent advances in CRISPR/Cas12a engineering for activity enhancement and exponential signal amplification, with a particular emphasis on crRNA, activator strand, reporter modulation and environmental regulation for catalytic activity enhancement or exponential signal amplification (Fig. 1). We discuss the underlying design principles, amplification mechanisms, representative applications, and remaining challenges associated with each strategy. By highlighting how intrinsic molecular engineering can transform Cas12a from a linear-response nuclease into a self-amplifying biosensing system, we aim to provide both a conceptual foundation and a practical reference for the future development of robust, sensitive, and field-deployable CRISPR/Cas12a diagnostic platforms.

## Programming CRISPR/Cas12a system components for activity enhancement and exponential signal amplification

### crRNA engineering: from passive recognition to active regulation for signal amplification

The CRISPR RNA (crRNA), also known as guide RNA, is a fundamental component of the CRISPR/Cas12a system. It directs the Cas12a protein to specific target sequences and initiates both *cis-* and *trans*-cleavage reactions.^72,73^ The efficiency of Cas12a's *trans*-cleavage activity, including its turnover rate, substrate accessibility, and activation kinetics, is strongly influenced by the spatial configuration, sequence properties, and terminal chemical environment of crRNA.^74^ Previous work has established that crRNA conformation plays a critical role in the assembly and function of the Cas12a/crRNA/activator complex.^75^ Consequently, strategic engineering of crRNA through structural design, sequence extension, or programmable modification can not only improve the turnover rate but also enable the construction of positive feedback loops, which transform the signal output from a linear mode to an exponential amplification process, offering a promising route to highly sensitive biosensing without the need for pre-amplification. This section systematically reviews recent advances in crRNA editing strategies for modulating CRISPR/Cas12a activity. By shifting the role of crRNA from a mere targeting guide to an active signal amplifier, researchers have achieved dynamic control over the system, markedly improving biosensor performance and expanding its applications.

### Terminal extension modifications of crRNA: for enhanced *trans-*cleavage activity

A typical crRNA consists of a direct repeat region, which binds the Cas12a protein and stabilizes the ribonucleoprotein complex, and a spacer sequence (guide sequence) that mediates target recognition.^76,77^ Both elements influence targeting accuracy and cleavage efficiency. Modifying the terminal regions (3′- or 5′-end) of crRNA has emerged as a common strategy to enhance Cas12a's *trans*-cleavage activity.^78,79^ For instance, the introduction of nucleic acid extensions alters the Cas12a/crRNA conformation, modulates activation kinetics, and enhances both target recognition and *trans*-cleavage activity, leading to improved signal amplification.

### 3′-end extension: targeting the catalytic core

**Fig. 2:**
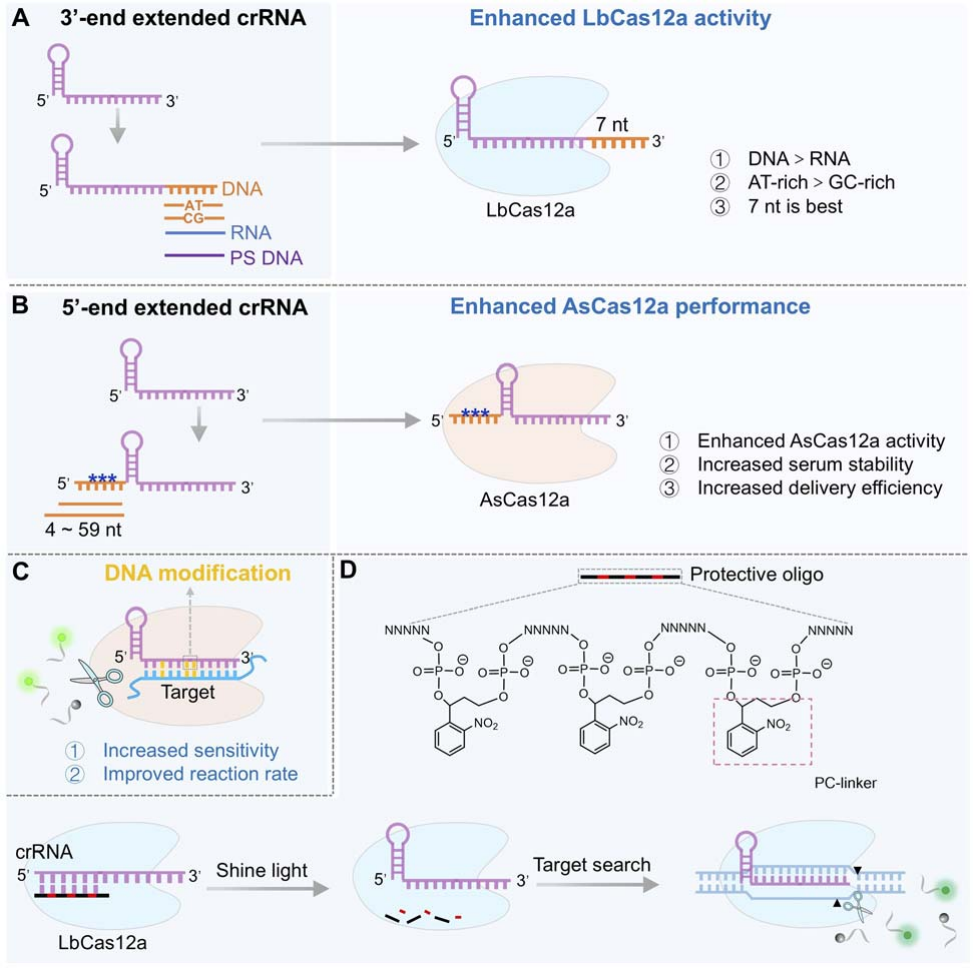
Engineering crRNA to enhance the performance of the CRISPR/Cas12a system. (A) Enhancement of LbCas12a *trans*-cleavage activity by a 3′-end ssDNA extension, the effect of which depends on the extension's length and nucleotide composition. (B) Improvement of AsCas12a *trans*-cleavage activity, stability, and delivery efficiency *via* a 5′-end ssRNA extension. (C) Boost in *trans*-cleavage activity through the incorporation of DNA bases into the crRNA spacer region. (D) Schematic of photoactivatable crRNA design enabling spatiotemporal, on-demand control of *trans*-cleavage activity.

Crystal structure analyses suggest that the 3′-end of crRNA is situated near the RuvC catalytic core domain of LbCas12a, and it was proposed that extending this terminal region could induce conformational changes that enhance the non-specific cleavage rates of ssDNA.^78^ Systematic evaluation of various extension types (ssDNA, ssRNA, and phosphorothioate (PS)-modified DNA) and lengths revealed that a 7-nt ssDNA extension at the 3′-end of crRNA enhanced *trans*-cleavage activity by 3.5-fold, with the ssDNA extension proving superior to the ssRNA extension and AT-rich sequences outperforming GC-rich ones (Fig. 2A). This modification also reduced background noise without compromising target binding, ultimately improving detection sensitivity from picomolar (pM) to femtomolar (fM) levels. Mechanistically, the 3′-end extension facilitates proximity-driven interactions with the RuvC domain of Cas12a, promoting catalytic site exposure and thereby increasing cleavage rates. Following target recognition, the extended sequence itself undergoes autocatalytic cleavage, generating free ends that further activate the sustained cleavage activity of Cas12a, thereby establishing a “cleavage–activation–recleavage” cascade effect. AT-rich extended sequences were found to be more effective than GC-rich ones, and this enhancement was consistent across different spacer sequences in LbCas12a. Notably, the length and chemical properties of the terminal extension are crucial for activity regulation.^80^ For instance, phosphorothioate modification at the 3′-end of the ssDNA extension significantly suppressed Cas12a activity. Collectively, these findings establish the 3′-terminal 7 nt AT-rich ssDNA extension as an optimal design balancing activity and specificity, which has been successfully applied in highly sensitive detection of pathogens such as SARS-CoV-2 and HIV.

### 5′-end extension: modulating recognition kinetics and complex stability

In contrast to 3′-end strategies that target the RuvC core, 5′-end extensions of crRNA primarily enhance Cas12a performance by improving the stability and delivery of the ribonucleoprotein complex, particularly in the context of AsCas12a. Structural studies indicate that the 5′-end of crRNA is exposed within the AsCas12a/crRNA complex, permitting rational engineering without disrupting protein–RNA interactions.^81^ Extension of ssRNA with 4 to 59 nucleotides at the 5′-end of crRNA has been shown to enhance Cas12a's *trans*-cleavage activity, with short extensions (4–9 nt) offering the best balance between activity and specificity (Fig. 2B). A key advantage of 5′-end extensions is their compatibility with chemical modifications, such as 2′-*O*-methylation, phosphorothioation, and deoxynucleotide substitutions. In contrast to the activity loss often caused by direct 5′-end modification of an unextended crRNA, the spatial flexibility afforded by an extension enables the incorporation of these modification groups without compromising cleavage efficiency. Moreover, 5′-end extensions significantly enhance the serum stability of crRNA and facilitate efficient delivery of Cas12a ribonucleoprotein (RNP) complexes *via* cationic carriers, underscoring their clinical utility. Supporting evidence comes from the “NEXT CRISPR” system, in which a 4-nt 5′-end extension of crRNA improved AsCas12a's *trans*-cleavage activity,^82^ enabling attomolar (aM)-level detection of Human Papillomavirus 16 (HPV16) DNA within 30 min using both fluorescence and lateral flow readouts. This enhancement is likely due to conformational changes in Cas12a protein, which promote non-specific cleavage of ssDNA.^81,83^ Overall, 5′-end engineering represents a versatile strategy for enhancing Cas12a performance across gene editing and molecular diagnostics.

### Chemical modifications: from stability to kinetic regulation

Beyond terminal extensions, chemical modification of crRNA provides another powerful avenue to enhance biosensor performance. By altering the phosphate backbone, nucleobase, or sugar moieties, these modifications can significantly improve crRNA stability, binding affinity, and specificity.^40,84–86^ Common modifications include base modifications (*e.g.*, locked nucleic acid (LNA), base analogues such as 5-bromo-2′-deoxyuridine and 2-amino-purine), backbone modifications (*e.g.*, 2′-*O*-methylation, phosphorothioate) and fluorescent labeling. These modifications increase resistance to nucleases, extend the half-life in complex matrices, and improve overall detection reliability.

In one notable example, an RNA-DNA hybrid crRNA was developed by replacing several RNA bases in the spacer region of crRNA with DNA bases (Fig. 2C), which enhanced base-pairing stability by reducing crRNA flexibility and thereby significantly improved both the target recognition efficiency and *trans*-cleavage activity of Cas12a.^87^ This strategy greatly increased the sensitivity of SARS-CoV-2 detection. Similarly, incorporating phosphorothioate modifications into the crRNA backbone at the 5′-end enhances resistance to intracellular nucleases and prolongs its half-life without compromising Cas12a activation, making it suitable for living cell and *in vivo* applications.^37^

Furthermore, the design of environmentally responsive crRNA through chemical modifications enables the on-demand activation of Cas12a, achieving precise spatiotemporal control for the construction of intelligent biosensors.^40,88^ A notable example is the development of a light-controlled crRNA.^89^ By incorporating a photocleavable cross-linker into its spacer region, the crRNA remains inactive until UV irradiation, upon which it is activated to trigger the detection signal, realizing “on-demand activation” (Fig. 2D). This strategy reduces background noise and improves the signal-to-noise ratio by 5–10 times, making it particularly suitable for detecting low-abundance targets in complex matrices such as serum or cell lysates. Importantly, this photoactivatable Cas12a system allows for one-pot combinatorial assays with other signal amplification methods, and may hold great promise for designing exponential amplification strategies. Upon coupling with other signal amplification techniques in a one-pot manner, the Cas12a reaction can be temporally controlled (activated by light only after other amplification steps are completed), thereby effectively preventing any interference from Cas12's non-specific cleavage activity during the upstream processes. Other modifications, such as 2′-*O*-methylation at the 3′-end of crRNA, can reduce off-target effects while preserving the high targeting affinity of the CRISPR/Cas12a system. This modification effectively suppresses non-specific binding interactions, further enhancing detection specificity.^90^

### Programmable crRNA switches: engineering autocatalytic feedback for exponential amplification

The development of programmable crRNA switches represents a paradigm shift toward dynamic control of Cas12a activity through self-catalyzed editing, enabling positive feedback loops for signal amplification. By incorporating programmable modules, such as stem-loop structures, aptamers or nucleic acid zymes, into the crRNA sequence, researchers have constructed self-sustaining “target recognition–switch activation–signal amplification” networks capable of exponential signal output.^91–94^ Central to this design is the exploitation of crRNA conformational changes to trigger sustained Cas12a activation.

**Fig. 3:**
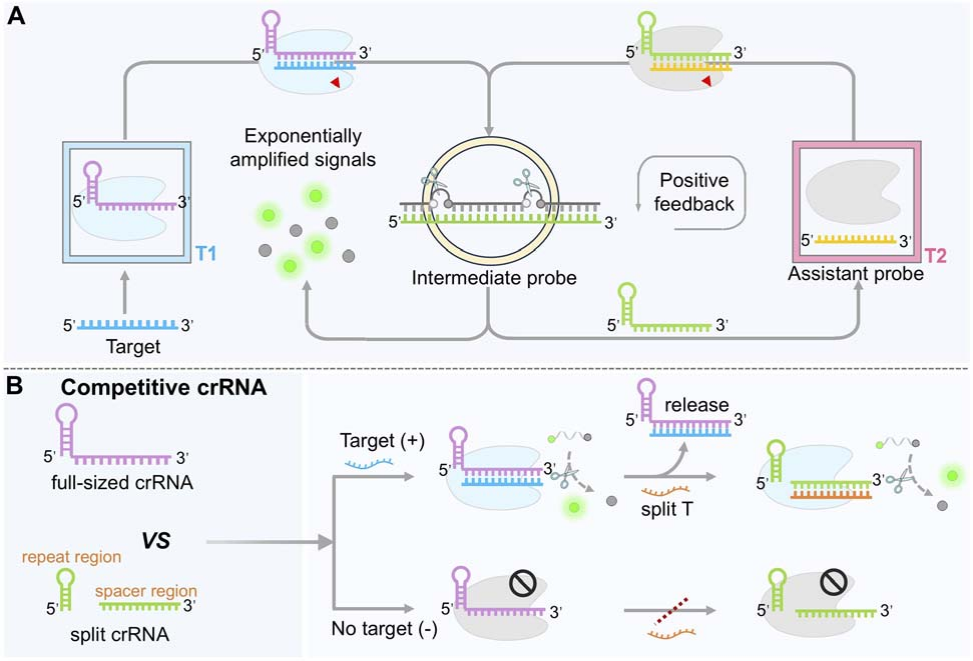
Engineering crRNA to construct autocatalytic and cascade amplification circuits. (A) Mechanism of the CRISPR/Cas12a autocatalysis-driven feedback amplification network (CONAN system). (B) Principle of cascade signal amplification based on competitive binding of Cas12a between full-length and split crRNAs.

A landmark example is the switchable-caged guide RNA (scgRNA) system, also known as CONAN (CRISPR-Cas-only amplification network) (Fig. 3A).^74^ This system employed two crRNA/Cas12a complexes. In the secondary complex (T2), the crRNA (green color) pre-hybridizes with an inhibitory DNA (iDNA) to form a stem-loop structure (intermediate probe) that maintains the crRNA in an inactive “off” state. Upon target-induced activation of the primary system (T1), its *trans*-cleavage activity cleaves the single-stranded loop region of the iDNA in the T2 system, liberating the crRNA and switching on Cas12a activity. The activated T2 complex then cleaves additional iDNA molecules, establishing an autocatalytic cycle that leads to an exponential increase in fluorescence signal. Under optimal experimental conditions, this single-enzyme detection method exhibits an approximately 363.8-fold enhancement in signal intensity compared with the conventional Cas12a linear amplification system. It achieves aM sensitivity and has been successfully applied to the detection of hepatitis B virus (HBV) and bladder cancer-associated gene mutations, with single-nucleotide discrimination specificity improved by 7.2–10.4 times.

### Splitting crRNA strategies: enabling competitive binding and cascade amplification

The splitting of crRNA into two or more fragments, such as separating the repeat region from the spacer region, offers another versatile route to enhance CRISPR/Cas12a functionality.^76^ Remarkably, Cas12a retains significant activity even when crRNA is split only between these two segments or cleaved at arbitrary positions within the spacer region. An exception occurs when the split is introduced between the 8th and 9th nucleotides at the 5′-end of the spacer region, which nearly abolishes CRISPR/Cas12a activity. This loss of function may be attributed to the disruption of key stabilizing interactions involving Lys897 and Lys900 in LbCas12a, which normally interact with the phosphate group between the 8th and 9th bases of the DNA activator. Cleavage at this specific site likely impairs protein–substrate binding.^95^ This splittable property allows the CRISPR/Cas12a system to be directly adapted for RNA detection by using the target RNA itself as the spacer component of a split crRNA. Upon hybridization with a complementary DNA activator, the reconstituted crRNA efficiently triggers *trans*-cleavage. This modular design not only improves biosensor flexibility, particularly for ultra-short RNA detection and multiplex analysis, but also enables discrimination between mature microRNAs (miRNAs) and their precursors (pre-miRNAs),^77,96–100^ significantly expanding the system's applicability and offering new opportunities for designing novel exponential signal amplification strategies.

To further improve sensitivity and versatility, split crRNA strategies have been integrated with competitive binding mechanisms. Studies have revealed that full-length crRNA and split crRNA exhibit distinct binding affinities for Cas12a.^101,102^ By co-introducing full-length crRNA and a non-homologous split crRNA, each designed to recognize a distinct activator strand, researchers established a sequential activation cascade (Fig. 3B). In this asymmetric CRISPR system, the target first activates the full-length crRNA/Cas12a complex. The authors proposed that, once the *trans*-cleavage activity of the full-length crRNA is activated, Cas12a protein is subsequently released and can then bind to the split crRNA, thereby activating its *trans*-cleavage activity and forming a cascading reaction. This “target-triggered, stepwise activation” mechanism amplifies the signal up to 1000-fold, enabling microRNA detection at concentrations as low as 856 aM without reverse transcription or pre-amplification, as demonstrated in clinical plasma samples from bladder cancer patients. The same principle has been adapted for electrochemical aptasensors (E-A-CRISPR), allowing ultrasensitive detection of small molecules such as clenbuterol (CLB),^103^ thereby extending the utility of CRISPR/Cas12a to non-nucleic acid targets.

The split crRNA strategy disrupts the conventional one-to-one target recognition and activation mode, replacing it with an artificially regulated “inactivation–reconstruction–amplification” cascade that supports exponential signal accumulation. Key advantages include low background, high programmability, compatibility with multimodal readouts, and broad target adaptability. A growing body of work confirms the considerable design flexibility offered by crRNA splitting, highlighting its potential for developing novel exponential signal amplification strategies.

In summary, crRNA engineering, through structural optimization, chemical modification, and programmable switch design, has transformed the CRISPR/Cas12a system from a linear signal generator into an exponential amplification platform. These advances enhance Cas12a performance on multiple fronts: improving enzymatic kinetics, enabling self-sustaining activation cycles, and diversifying reaction modalities. Notably, the effectiveness of these engineering strategies is not universally conserved across different Cas12a orthologs. For example, LbCas12a generally exhibits higher tolerance to terminal extensions and chemical modifications, often resulting in enhanced *trans*-cleavage activity, whereas AsCas12a is more sensitive to structural perturbations of crRNA and therefore requires more precise optimization. In contrast, MeCas12a displays distinct ion-dependent catalytic behavior, which can further modulate the impact of crRNA design. These ortholog-dependent differences highlight that rational crRNA design should not be viewed as a universally transferable strategy, but rather as one that requires ortholog-specific evaluation and optimization. Despite these significant advances, challenges remain in delivery efficiency, multi-target compatibility, and standardization. Nevertheless, the ongoing integration of nanotechnology, artificial intelligence, and microfluidics is expected to further accelerate the translation of CRISPR/Cas12a biosensors from laboratory research to clinical POCT testing, establishing them as important tools in next-generation molecular diagnostics.

### Activator strand engineering: from “one-target-one-enzyme” to autocatalytic signaling networks

Within the CRISPR/Cas12a system, the activator strand serves as the critical link between target recognition and the enzyme's *trans*-cleavage reaction. It not only determines recognition specificity but also acts as a central “switch” for signal amplification.^104,105^ Conventionally, Cas12a activator strands have been limited to ssDNA and dsDNA, a lack of diversity that has constrained the functional versatility and broader application of the system.^23,60,106^

Traditional biosensor designs follow a “one-target-one-enzyme” model, where a single target molecule activates only one Cas12a/crRNA complex, resulting in limited *trans*-cleavage events and linear signal amplification. This inherent limitation hinders the detection of low-abundance biomarkers. Recent research has therefore focused on directly engineering the activator strand itself, including modulating its length, structure, topology, chemical modifications, and reconfigurability, to regulate its binding mode with Cas12a, cleavage kinetics, and regenerative capability. By constructing positive feedback loops, the activator strand can be upgraded from a “passive trigger” to a “synergistic amplifier”, enabling exponential signal output. The following sections analyze and summarize the structural designs, modification strategies and underlying mechanisms of these engineered activator strands.

### Topological regulation: circular-linear conversion-mediated autocatalytic feedback

**Fig. 4:**
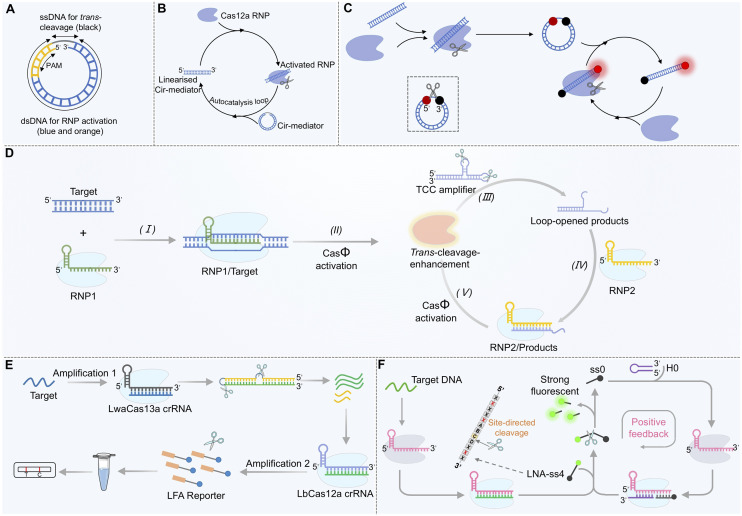
Engineering activator strands to construct autocatalytic and cascade amplification circuits for the CRISPR/Cas12a system. (A) Schematic of the Cir-mediator capable of switching between inactive (cyclic) and active (linearized) states. (B) Mechanism of the Cas12a autocatalytic feedback loop initiated by Cir-mediator linearization. (C) Fluorescence signal generation principle using the cleaved Cir-mediator as both the activator and reporter. (D) Autocatalytic amplification system mediated by a DNA activator with two stem-loop structures. (E) Cas13a-Cas12a cascade activation strategy using the RNA-DNA hybrid activator. (F) Working principle of the LNA-modified split activator system (CALSA) for self-sustaining amplification.

Research has shown that the activation efficiency of Cas12a can be profoundly influenced by altering the structure and sequence of the activator strand.^107^ A groundbreaking study in 2024 reported that short DNA with a circular topological structure (Cir-mediator) is virtually incapable of activating the Cas12a/crRNA complex due to its constrained spatial conformation (Fig. 4A).^58^ This Cir-mediator consists of a dsDNA region (containing both a protospacer adjacent motif (PAM) sequence and a target sequence complementary to the crRNA) and a ssDNA linker. Once this circular structure is linearized (for instance, through cleavage of its ssDNA linker by activated Cas12a), the exposed dsDNA region becomes an efficient activator that can activate additional Cas12a proteins. This initiates an exponential *trans*-cleavage reaction characterized by “circular shielding–cleavage-induced linearization–sustained activation” (Fig. 4B). By labeling the circular structure with a fluorophore and a quencher at opposite ends, exponential amplification of the fluorescence signal can be achieved (Fig. 4C). Herein, the Cir-mediator plays two critical roles simultaneously: activator and reporter. This system enables aM-level detection of both DNA and RNA without pre-amplification, and notably, RNA detection does not require reverse transcription. The signal growth approximates an exponential curve while maintaining low background noise.

In a complementary approach (Fig. 4D), an activator strand containing two stem-loop structures was designed for use with CRISPR/Cas*Φ* (a Cas12 family member).^108^ In this configuration, once the *trans*-cleavage activity of the first CRISPR/Cas*Φ* system is activated by the target, it cleaves the loop regions of the two stem-loop structures, releasing an activator strand with a 3′-toehold that is highly efficient at activating a second CRISPR/Cas*Φ* system. The activated secondary system then cleaves additional stem-loop structures, releasing more activators and establishing a “cleavage–activation–re-cleavage” cascade amplification effect. This mechanism, which relies on “structural inhibition–enzyme cleavage unlocking–cascade activation”, uses the activator strand as a delayed-release secondary trigger, effectively amplifying the signal while controlling the background. This strategy achieved ultra-sensitive detection of pathogen DNA (detection limit as low as 0.11 copies per µL) and demonstrated the ability to detect pathogens at concentrations as low as 1.2 CFU mL^−1^ in serum samples within 40 min, outperforming qPCR in sensitivity.

The combination of Cas13a and Cas12a allows for the construction of a cascade signal amplification system. In this design, the Cas12a activator is engineered as an RNA-DNA hybrid (Fig. 4E). When the target RNA activates Cas13a's *trans*-cleavage activity, Cas13a cleaves the RNA component (blue) of the hybrid, releasing a pre-locked single-stranded DNA trigger (green) from a hairpin structure. The released ssDNA then serves as the activator strand for Cas12a, generating a detectable signal.^53^ This process achieves cascade conversion and signal amplification from RNA recognition (Cas13a) to DNA-based amplification (Cas12a), lowering the detection limit to 1 aM and enabling discrimination among different SARS-CoV-2 variants. The core mechanism involves using stem-loop structures to reduce background signals *via* steric hindrance, while the cleaved linear fragments act as new activator strands to initiate the cascade reaction, enabling efficient signal accumulation.^60,109–111^

### Split activators and collaborative amplification: from monomeric to synergistic activation

Similar to crRNA, the activator strand can also be split into two segments, neither of which alone can effectively activate *trans*-cleavage activity but which can restore function upon combination.^51,55,112^ The “split-coassembly” strategy involves dividing the activator into functionally complementary subunits that undergo secondary assembly within the Cas12a RNP complex, upgrading the process from linear activation to exponential amplification. Such designs can be extended to construct “AND” logic gates or multi-input responsive circuits, significantly enhancing the specificity for single-nucleotide polymorphism detection and offering new avenues for disease genotyping.

Based on this concept, a strategy termed CRISPR/Cas autocatalysis amplification driven by LNA-modified split activators (CALSA) was developed.^56^ In this design, a 12-nt ssDNA serves as the *trans*-cleavage substrate. Introducing LNA modifications at specific sites within this substrate enables activated LbCas12a to cleave it into two defined fragments. One of these fragments, when combined with a hairpin DNA, can activate a second CRISPR/Cas12a system. The activated secondary system then established a positive feedback loop by cleaving more 12-nt ssDNA substrates, which in turn activated more CRISPR/Cas12a complexes, forming an “activation–cleavage–reactivation” cycle (Fig. 4F). When the 12-nt ssDNA is labelled with a fluorophore-quencher pair at its termini, exponential amplification of the fluorescence signal is achieved, with the ssDNA functioning dually as both an activator and a reporter probe. This system reduces the detection limit for the BRCA-1 gene to 4.7 fM and can distinguish single-base mutations, showing promise for early detection of circulating tumor DNA (ctDNA). Future directions may involve transferring “controllable activation” logic from crRNA to the activator strand, potentially simplifying the system, shortening reaction time, and enabling dynamic tracking of target appearance and disappearance. Activator strand engineering is thus evolving from “single-structure optimization” to “modular collaborative design”.

In addition to the specific design mentioned, activator strands can be further modified or engineered to enhance the flexibility and performance of CRISPR/Cas12a-based biosensors. Strategies such as splitting, RESET effects, and structural modifications expand their applicability to direct RNA detection, cellular imaging, and single-nucleotide variant discrimination.^112–114^ These modifications not only increase detection sensitivity and specificity but can also boost enzymatic activity and enable exponential signal amplification schemes, providing versatile design principles for next-generation nucleic acid diagnostics.

In summary, the activator strand is the core mediator connecting target recognition and signal output in the CRISPR/Cas12a system. Precise editing of its structure and function is key to overcoming the system's inherent signal amplification bottleneck. Through design strategies such as topological regulation, splitting, chemical modification, and modular synergy, “inhibition–unlocking–cascade” signal amplification networks have been constructed. These have improved the detection sensitivity of the CRISPR/Cas12a system from the nM to the aM level without external pre-amplification. The commonality among these methods lies in leveraging the structural plasticity of the activator strand to break the “one-target-one-enzyme” activation paradigm, achieving exponential signal amplification through self-catalytic cycles. Notably, circular, split, and stem-loop activator architectures share a unified design principle centered on rigorous background suppression to ensure high-fidelity activation. Circular activators employ topological constraints to physically block activator-Cas12a recognition and prevent spontaneous conformational activation, thereby eliminating leakage from partial strand invasion or non-specific binding. Split activators rely on modular separation to disrupt the structural integrity required for Cas12a engagement; individual fragments lack sufficient recognition elements and cannot trigger *trans*-cleavage unless spatially assembled, thus minimizing false activation. Stem-loop activators use intramolecular stem hybridization to sequester functional motifs and mask PAM or seed regions, creating a kinetic barrier that prevents premature Cas12a loading. Together, these mechanisms synergistically suppress stochastic cleavage, non-specific activation, and signal leakage, enabling the low-background operation critical for reliable exponential signal amplification in complex biosensing environments. Future efforts should focus on optimizing the stability of activator strands in complex matrices and developing multi-target parallel detection strategies. With the emergence of more intelligent designs (*e.g.*, environment-responsive and multivalent synergistic activators), the CRISPR/Cas12a system is expected to find broader applications in POCT diagnostics, multiplex detection, and *in vivo* imaging, providing core tools for early disease screening and precision therapy.

Despite the significant advantages of these autocatalytic and positive feedback designs, a critical challenge is the trade-off between suppressing the background (signal leakage) and maintaining efficient exponential amplification. In systems such as those illustrated in Fig. 4, unintended background activation can arise from incomplete structural inhibition, stochastic cleavage events, or transient exposure of activator domains, which may prematurely trigger feedback loops and compromise detection fidelity. To address this challenge, several strategies have been explored, including enhancing the stability of inactive states through rational structural design (*e.g.*, circular or stem-loop configurations), introducing kinetic barriers *via* multi-step activation or toehold-mediated processes, and implementing spatiotemporal control mechanisms such as photoactivation or phase separation. In addition, careful optimization of reaction conditions, including ion concentration and enzyme loading, can further suppress nonspecific activation while preserving amplification efficiency. These approaches collectively enable a more controlled transition from a low-background state to a high-gain amplification regime, thereby improving the robustness and reproducibility of exponential CRISPR/Cas12a systems.

### Reporter probe engineering: from background suppression to signal recycling and exponential output

As the final carrier of signal output, the design and composition of the reporter probe directly determine the signal transduction efficiency, background noise level and overall detection sensitivity of a CRISPR/Cas12a system.^115^ Conventional reporter probes are typically short linear ssDNA molecules labeled with a fluorophore (*e.g.*, FAM) and a quencher (*e.g.*, BHQ) at their termini (termed FQ-Reporter). Upon Cas12a activation, its *trans*-cleavage activity non-specifically cleaves these probes, separating the fluorophore from the quencher and generating a fluorescent signal.^116^ However, this traditional linear FQ-Reporter system is constrained by the “one-target-one-enzyme” activation mode, where each activated Cas12a cleaves only a limited number of probes. Additional limitations include high background noise, susceptibility to degradation, photobleaching, insufficient quenching efficiency, and poor photostability, which collectively cap further improvements in detection sensitivity.

In recent years, engineering the reporter probe itself has emerged as a pivotal strategy for enhancing the signal amplification capability of Cas12a. Beyond extending the length of the reporter strand (from 5 nt to 30 nt or longer) to overcome the limitation that Cas12a cannot be directly used for RNA detection,^117,118^ researchers have achieved significant breakthroughs through precise chemical modifications, structural redesign (*e.g.*, incorporating hairpin or circular conformations), and coupling with nanomaterials. These innovations have upgraded the signal output from a linear “one-to-one” mode to an “one-to-many” or even a “self-recycling” exponential amplification system.^20,119^ By overcoming the limitations of traditional FQ-Reporters, these advanced probes offer superior sensitivity, interference resistance, and device compatibility, thereby expanding the application scope of CRISPR/Cas12a-based detection.

### Chemical modifications and signal transduction optimization: from “one-target-one-light” to “one-target-multiple-light”

**Fig. 5:**
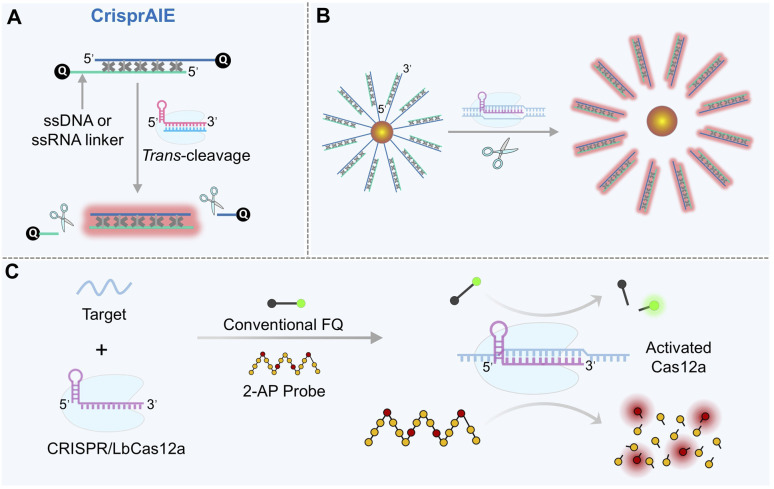
Advanced reporter probes for enhanced signal output in the CRISPR/Cas12a system. (A) Schematic of the “CrisprAIE” sensing principle using AIEgens. (B) Sensing mechanism of the SNA-AIEgen reporter. The polydopamine and AuNP-coated Fe_3_O_4_ nanocomplex (Fe_3_O_4_/Au/PDA) acts as the nanoquencher of AIEgens. (C) Comparison of the CRISPR/Cas12 system using a conventional single-stranded FQ-Reporter *versus* a quencher-free 2-AP probe.

Introducing advanced chemical motifs, such as aggregation-induced emission (AIE) luminogens (AIEgens), can surpass the signal ceiling of the conventional fluorescence quenching-release mode. AIEgens exhibit weak fluorescence in free states but emit intense fluorescence upon aggregation or spatial confinement, offering large Stokes shifts and excellent photostability.^120^ Leveraging these properties, a novel probe termed “CrisprAIE” was developed.^121^ This system employs a dsDNA reporter with single-stranded overhangs, each terminally labeled with a quencher. Cationic AIEgens (*e.g.*, 4′,4′′,4′′′-(((((1*E*,1′*E*)-benzo[*c*][1,2,5]thiadiazole-4,7-diylbis(ethene-2,1-diyl))bis(4,1-phenylene))bis(azanetriyl))tetrakis(benzene-4,1 diyl))tetrakis(1-methylpyridin-1-ium) iodide (TPBT)) intercalate into the dsDNA grooves, where their fluorescence is initially quenched by the terminal quenchers. Upon CRISPR/Cas12a activation, cleavage at the single-stranded overhangs removes the quenchers, allowing the embedded AIEgens to emit strong fluorescence (Fig. 5A). Since multiple AIEgen molecules can be incorporated into each dsDNA reporter, this design enables a “one-to-many” signal output (a single cleavage event releases numerous fluorescent molecules), achieving 80–270-fold higher signal gain than traditional FQ-Reporters and lowering the detection limit to fM or even aM levels. Further coupling with spherical nucleic acids (SNAs) enhanced stability and clinical utility (Fig. 5B), enabling successful detection of norovirus and SARS-CoV-2 in patient samples. AIEgen-based reporters are also compatible with various CRISPR/Cas detection platforms (*e.g.*, SHERLOCK,^122,123^ DETECTOR,^124^ and HOLMES^125^), significantly boosting their sensitivity and robustness.

Quencher-free reporter probes based on environment-responsive molecules offer an alternative pathway for sensitivity enhancement. A prominent example is the 2-aminopurine (2-AP) probe (Fig. 5C).^126^ In its free state, 2-AP exhibits strong fluorescence due to unrestricted intramolecular motion. However, when incorporated into ssDNA, its fluorescence is suppressed by base stacking and the local polar environment, and it is nearly completely quenched upon integration into dsDNA. This environment-dependent fluorescence change eliminates the need for a separate quencher, avoiding the synthetic complexity and potential background interference associated with traditional FQ-Reporter probes. Unlike the “single cleavage–single signal” mode of FQ-Reporter probes, a single ssDNA can embed multiple 2-AP molecules (*e.g.*, four). Thus, one Cas12a cleavage event can release several fluorescent units. 2-AP probes are particularly suitable for detecting low-abundance viral nucleic acids (*e.g.*, goat pox virus (GTPV) and HPV) and ctDNA, offering a cost-effective and operationally simple solution for pathogen screening and early cancer detection in resource-limited settings.

### Nanomaterial-coupled reporter probes: from local field enhancement to multilevel amplification and cross-modal transduction

Immobilizing reporter probes onto nanocarriers to prepare SNAs represents an effective strategy for signal enhancement by regulating probe density, spatial distribution, and nuclease resistance.^64^ For instance, constructing a high-density, aligned nucleic acid shell on gold nanoparticles (AuNPs) or Au nanostars (AuNSTs) significantly improves the biological stability of reporters and optimizes fluorescence signal transduction.^127–129^ AuNPs themselves act as efficient fluorescence quenchers. When fluorescently labeled reporters are adsorbed onto AuNP surfaces, fluorescence is quenched. Target-activated Cas12a cleavage releases short fluorescent fragments, restoring fluorescence (“signal-on” state).

The core of nanomaterial-based signal enhancement lies in the “local field enhancement” effect. This phenomenon is driven by the surface plasmon resonance (SPR) of noble metal nanoparticles, which generates intensified electromagnetic fields near the particle surface, thereby amplifying the signal from adjacent fluorophores or Raman tags. Realizing this effect requires precise chemical modification to control the spatial distance and surface charge of the nanoparticles. It is essential to position signal molecules within the SPR “enhancement zone” (typically within 5–10 nm of the nanoparticle surface), while preventing nanoparticle aggregation or nonspecific adsorption that could undermine signal fidelity.^130,131^ A central strategy for achieving such control involves the chemical modification of gold nanoparticles, with thiol-modified ssDNA of a defined sequence anchored to the gold surface. This configuration serves a dual purpose: the ssDNA acts as the *trans*-cleavage substrate for Cas12a, and upon target-induced activation, cleavage of the ssDNA alters the surface conformation, effectively modulating the distance between the signal molecule and the gold nanoparticles. Simultaneously, the negatively charged ssDNA backbone provides electrostatic repulsion that suppresses particle aggregation, thereby maintaining colloidal stability and preserving the SPR enhancement effect.

**Fig. 6:**
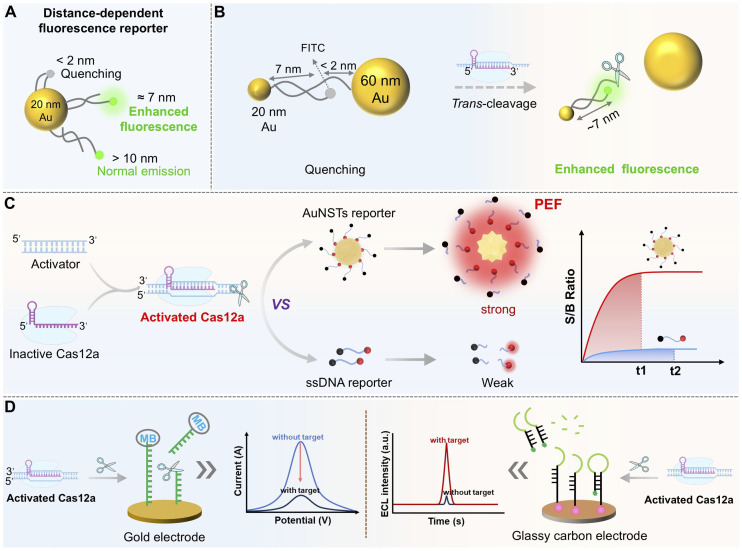
MEF effect or SNA-enhanced fluorescence and electrochemical readouts for CRISPR/Cas12a-based sensing. (A) Diagram of the effect of the dsDNA length on the MEF effect between a 20 nm AuNP and FITC. (B) Mechanism of MEF-based signal amplification using AuNP nanosensors upon Cas12a *trans*-cleavage activation. (C) Performance comparison between conventional ssDNA reporters and SNA reporters in CRISPR/Cas12a-based sensing systems. (D) Working principles of electrochemical and electrochemiluminescence signal transducers for CRISPR/Cas12a-based detection.

A representative study utilized dual-sized AuNPs (20 nm and 60 nm) assembled into a reporting probe *via* a bridging fluorescein isothiocyanate (FITC)-labeled dsDNA-ssDNA construct^26^ (Fig. 6A and B). In the absence of a target, FITC fluorescence is quenched due to proximity (<2 nm) to the 60 nm AuNP. Upon Cas12a activation, the ssDNA bridge is cleaved, releasing FITC from the 60 nm AuNP while maintaining a linkage to the 20 nm AuNP. By fine-tuning the FITC-20 nm AuNP distance to ∼7 nm using dsDNA, a metal-enhanced fluorescence (MEF) effect is triggered, resulting in amplified fluorescence and a visible color change from purple to reddish-purple. This approach enabled amplification-free detection of BRCA-1 within 30 min at fM sensitivity, exemplifying a “self-amplification” design where Cas12a cleavage modulates the spatial relationship between nanoparticles and signal molecules.

Building on this, a subsequent study combined nanoconfinement with plasmon-enhanced fluorescence (PEF) by employing AuNSTs featuring 20 symmetrical “hot spots” coated with a tunable silica shell (Fig. 6C).^132^ Cy5.5/BHQ-labeled ssDNA probes were anchored to the surface. The silica shell adjusted the optimal fluorophore-metal distance, achieving a ∼21.5-fold enhancement in the signal-to-background ratio. Spatial confinement increased the collision frequency between Cas12a and the substrate, reducing the cleavage time from 35 to 15 min and maintaining high efficiency even at low intracellular Mg^2+^ concentration (∼1 mM). This probe detected miRNA-375 and prostate-specific antigen (PSA) at limits of 17.3 fM and 27.7 pg mL^−1^, respectively, representing ∼196-fold and ∼122-fold improvements over conventional ssDNA probes. SNA- or PEF-type reporters not only enhance stability and signal-to-noise ratios but also establish new paradigms for Cas12a applications in complex systems and living cells.

By systematically investigating Cas12a's *trans*-cleavage activity on the AuNP surface,^133^ it was demonstrated that LbCas12a retained high cleavage efficiency on SNA shells, where the densely packed nucleic acid architecture confers remarkable probe stability and protects against nuclease degradation in serum, substantially reducing false-positive backgrounds. The large surface area of AuNPs allows high-density loading of reporters, enabling a single Cas12a activation event to release numerous fluorophores for primary signal amplification (Fig. 6D). By optimizing DNA density and length, the detection limit was reduced to 10 fM, approximately two orders of magnitude better than that of traditional ssDNA probes, with stable performance in 100% serum. Integrating nuclease-resistant SNA reporters with rolling circle amplification (RCA) products created an RCA-Cas12a cascade system, achieving a detection limit of 10 aM for ctDNA.^134^ These findings underscore the exceptional stability and minimal background drift of SNA-based probes in complex biofluids, highlighting their strong potential for clinical liquid biopsy applications.

The core design principle of modulating the spatial relationship between nanomaterials and signal molecules through Cas12a-mediated cleavage can be extended to electrochemical detection, forming a unified framework for multimodal signal transduction.^135^ In classic electrochemical CRISPR (E-CRISPR) platforms, thiolated ssDNA substrates labeled with an electrochemical label (*e.g.*, methylene blue (MB) and ferrocene) are immobilized on a gold electrode.^15,136,137^ Target-activated Cas12a-catalyzed ssDNA cleavage separates the tag from the electrode surface or alters its electron transfer distance, causing a measurable change in faradaic current detectable by differential pulse voltammetry (DPV), electrochemical impedance spectroscopy (EIS) or electrochemiluminescence (ECL) measurement. This shift from a “photon” to “electron” readout allows the same cleavage event to be monitored *via* both optical and electrochemical modes, enhancing result reliability and enabling the development of robust, multimodal CRISPR/Cas12a detection platforms for POCT (Fig. 6D).^138^

### Structured reporter probes: from single-event cleavage to positive feedback loops

Beyond traditional linear FQ-Reporters, a variety of structured reporter probes have been designed to further enhance the signal amplification capabilities of the CRISPR/Cas12a system. One of the most impactful designs is the hairpin-shaped molecular beacon probe, which consists of a complementary stem region and a single-stranded loop. The stem termini are typically modified with a fluorophore and a quencher (*e.g.*, FAM/BHQ1), while the loop serves as the *trans*-cleavage substrate for Cas12a. The key advantage of this configuration is the spatial proximity between the fluorophore and quencher in the intact state, enabling efficient fluorescence resonance energy transfer (FRET) that significantly reduces the initial background noise.^66,139,140^ Furthermore, the unique conformation of hairpin DNA enhances its affinity for the Cas12a catalytic pocket. Michaelis–Menten analysis has confirmed that hairpin reporters exhibit lower *K*_m_ values compared to linear ssDNA reporters, indicating stronger enzyme–substrate binding. Through precise secondary structure design, hairpin probes have achieved integrated breakthroughs in structural optimization, activity regulation, and signal amplification. By functionally repurposing the cleavage products to establish positive feedback loops, these probes enable a transition from linear to exponential signal output without relying on exogenous nanomaterials or complex chemical modifications. Recently, researchers have focused on tuning secondary structure parameters (*e.g.*, stem length, loop size, and base composition), surface/interface modifications, and the construction of cyclic/feedback systems, which provides a new idea for the construction of cyclic/feedback systems to develop highly sensitive, upstream pre-amplification-free CRISPR/Cas12a sensing platforms.

### Structural parameter optimization

**Fig. 7:**
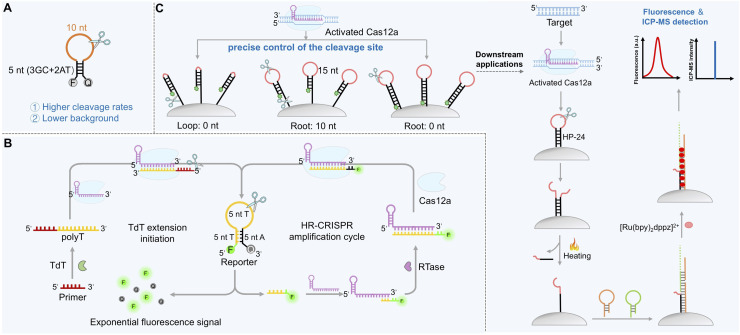
Harnessing structured DNA reporters for enhanced CRISPR/Cas12a-based sensing. (A) Influence of stem length, loop length, and base composition on the *trans*-cleavage activity of LbCas12a toward hairpin DNA reporters. (B) Working principle of the HR-CRISPR feedback amplification circuit for detecting TdT activity. (C) Schematic of a MB-immobilized hairpin DNA interface for a spatially regulated CRISPR/Cas12a cleavage reaction.

The performance of hairpin reporters is highly dependent on precise control over structural parameters. Stem stability directly influences the activation threshold: overly short stems (<5 bp) result in structural relaxation and an elevated background, whereas excessively long stems (>8 bp) hinder product release and slow signal generation. Nevertheless, it does not represent a fixed value, instead, it constitutes a “design window” in which a balance must be achieved between stability (low background) and accessibility/kinetic performance (high sensitivity and fast response). Similarly, loop length must balance cleavage efficiency and probe stability. Systematic studies have shown that a moderate loop length (*e.g.*, 10 nt) enhances binding to the Cas12a catalytic center, leading to higher cleavage rates while maintaining structural stability and a low background (Fig. 7A).^119^

### Feedback-driven hairpin systems

While conventional hairpin probes primarily operate in the “one-cleavage–one-release” linear mode, recent designs incorporate feedback mechanisms for exponential signal accumulation. One innovative example is the hairpin-driven, reverse transcriptase-assisted CRISPR/Cas12a positive feedback loop (HR-CRISPR).^141^ In this system, target-activated Cas12a cleaves an FQ-labeled hairpin probe, and one of the released fragments binds to the crRNA and is extended by reverse transcriptase to generate a sequence that activates secondary CRISPR/Cas12a complexes, establishing a product-driven autocatalytic cycle (Fig. 7B). This system achieved ultrasensitive detection of terminal deoxynucleotidyl transferase (TdT) activity (4.55 × 10^−6^ U) without pre-amplification and was successfully used to monitor TdT activity and screen inhibitors in human serum samples. Compared with traditional linear signal amplification, this HR-CRISPR system achieves exponential signal accumulation while maintaining a low background.

### Interface-immobilized hairpin reporters

In addition to the chemical modification, immobilizing hairpin probes on nanomaterial surfaces to construct three-dimensional reaction interfaces represents another effective strategy for improving sensing performance.^142,143^ For instance, anchoring biotin-labeled hairpin DNA onto magnetic beads (MB-hairpin interface) allows precise regulation of cleavage sites by adjusting the length of the “root” and “stem” regions.^66^ Extending the root to 10 nt reduced steric hindrance and increased the probability of Cas12a cleavage in the root region, whereas a root length of 0 nt confined cleavage primarily to the loop, enabling controllable cleavage positioning (Fig. 7C). This approach not only addresses fundamental issues of the background and reaction kinetics through hairpin structure and magnetic bead immobilization, but also facilitates seamless integration between CRISPR/Cas12a *trans*-cleavage and downstream dynamic DNA amplification techniques (*e.g.*, hybridization chain reaction, HCR), establishing a new paradigm for multifunctional and multimodal detection. This strategy demonstrates that integrating hairpin-structured reporters into dynamic DNA amplification networks can overcome the amplification limitations inherent to single-enzyme reactions. The integrated design concept of “CRISPR precise cleavage + downstream cascade amplification + multi-mode readout” establishes a novel pathway and provides a theoretical foundation for developing next-generation CRISPR biosensors with high performance and high reliability.

### Integration with non-canonical nucleic acid structures: development of label-free strategies

Recent efforts have gone beyond single stem-loop designs by incorporating non-classical nucleic acid structures, such as G-quadruplex (G4) and G-triplex (G3), to further break through the signal conversion ceiling of Cas12a in pre-amplification-free detection. In addition, one key advantage of these structures lies in enabling label-free detection, where the signaling dye remains free in solution rather than being covalently attached to the reporter probe.

G4 structures, formed by G-rich sequences, can specifically bind some small-molecule dyes (*e.g.*, *N*-methyl mesoporphyrin IX, NMM) and enhance their fluorescence by 100–1000 fold.^144^ This property is harnessed in a label-free CRISPR/Cas12a detection strategy (G-CRISPR-Cas) using a G-quadruplex probe, where signal transduction is achieved through specific G4-NMM binding.^145^ Upon Cas12a activation by LAMP amplicons, the G4 structure is cleaved, disrupting NMM binding and leading to a decrease in fluorescence intensity. This dual amplification system (LAMP + Cas12a) detected *Salmonella enterica* at a sensitivity of 20 CFU and enabled monitoring of bacterial colonization in chickens.

G3 structures, also formed by G-rich sequences, are the triple-stranded intermediates between ssDNA and G4; they exhibit favorable cleavage kinetics but require specific K^+^ concentrations for stability, which may conflict with the salt sensitivity of Cas12a.^146^ To resolve this, a compatible reaction environment was engineered using a “multi-crRNA cooperative recognition + buffer optimization” strategy, where low concentration of K^+^ was supplemented with Mg^2+^/Ca^2+^/PEG200 for co-stabilization.^147^ Under these conditions, a terminally labeled G-triplex probe demonstrated higher signal transduction efficiency than linear probes, achieving fM-level detection limits. This approach highlights the potential of combining high-affinity nucleic acid structures with precise environmental control to achieve synergistic improvement through enhanced catalytic site accessibility and substrate conformation matching.

Collectively, diverse reporter architectures exhibit distinct characteristics tailored for specific sensing demands. AIE-based reporters feature strong fluorescence intensity, large Stokes shifts, and high photostability, making them ideal for ultra-sensitive and visual detection; yet they require sophisticated probe design and fluorogenic element synthesis. Quencher-free 2-AP probes offer simple configuration, low background, and cost-effectiveness, making them suitable for rapid and high-throughput screening; however, their fluorescence quantum yield is relatively moderate. Hairpin reporters provide significantly suppressed background and enhanced substrate affinity *via* a stem-loop structure, supporting high-fidelity and feedback-driven amplification, but demand precise tuning of stem and loop lengths. G-quadruplex reporters enable label-free detection through specific dye recognition, which is advantageous for low-cost and simplified sensing systems, while their performance is susceptible to ionic conditions and sequence constraints. Nanomaterial-coupled reporters achieve remarkable signal enhancement *via* metal-enhanced fluorescence or surface confinement effects, exhibiting excellent anti-interference ability in complex matrices, yet they involve nanomaterial preparation and surface modification steps. This comparative overview provides a rational framework for selecting and designing optimal reporter probes to balance sensitivity, background, simplicity, and robustness in CRISPR/Cas12a-based biosensing.

In summary, the reporter probe serves as the core interface module for “target recognition–signal output” in the CRISPR/Cas12a system. Its evolution has consistently followed a logic centered on transitioning from “pursuing ultimate sensitivity” to “balancing practicality and clinical value”. Starting from initial linear signal output based on fluorescence quenching and release, the technology has matured into a diversified system combining high sensitivity, strong interference resistance, and excellent application compatibility. This development has progressed through stages of “linear–hairpin–non-classical conformation” in probe design, “exogenous dependence–self-circulation–multimodality” in the signal amplification strategy, and “laboratory–complex samples–POCT” in application scenarios. Each breakthrough has revolved around reducing background noise, improving efficiency, and simplifying operations.

As a signal amplification strategy for highly sensitive sensing, it is noteworthy that most currently reported pre-amplification-free CRISPR/Cas12a-based exponential signal amplification strategies involve the engineering and high-value utilization of reporter probes. In such systems, reporter probes not only function as signal output elements but also frequently perform additional roles. For instance, in Fig. 3A, the reporter probe serves simultaneously as a blocking strand that prevents crRNA from interacting with Cas12a and the activator strand. In Fig. 4C, the reporter probe forms an integral component of the crRNA double-stranded activator, cooperatively initiating Cas12a *trans*-cleavage activity. Furthermore, in Fig. 4F and 7B, the cleaved activator strand is repurposed either as a full activator or as part of a split activator to sustain CRISPR/Cas12a activation and enable autocatalytic amplification. Collectively, these multifunctional designs significantly enhance detection sensitivity, response speed, and amplification efficiency. Therefore, within exponential amplification frameworks, reporter probes frequently assume multiple roles, with their engineering profoundly influencing not only signal readout but also the regulation and propagation of amplification cascades.

Despite significant advances, challenges remain in environmental adaptability, sample stability, and multi-target compatibility. Future developments, such as intelligent response design, biomimetic protection strategies, and orthogonalization approaches, are expected to further promote the translation of CRISPR/Cas12a biosensors from laboratory research to clinical use. These tools hold great promise for providing low-cost, highly reliable detection in areas including pathogen detection, early tumor screening, and *in vivo* imaging, particularly benefiting public health prevention and control in resource-limited settings.

### External ions and reaction environment control: unlocking the kinetic potential of CRISPR/Cas12a

Beyond engineering the core molecular components (crRNA, activator strand, and reporter probe), the *trans*-cleavage activity of the CRISPR/Cas12a system is also profoundly influenced by external factors such as ion species, ion concentration, and buffer composition.^148,149^ As a metal-dependent nuclease, the conformation and catalytic efficiency of Cas12a's RuvC domain are strictly dependent on divalent metal ions (*e.g.*, Mg^2+^ and Mn^2+^).^150^ These ions participate in DNA unwinding, RuvC domain activation, and phosphodiester bond hydrolysis, thereby directly shaping both the cleavage efficiency and specificity of the system. Consequently, the optimization of metal ions and other environmental conditions has emerged as a powerful and straightforward strategy to enhance the reaction rate and detection sensitivity of Cas12a.

### Metal ions: concentration-dependent specificity “switch” and highly efficient activity enhancers

Mg^2+^ serves as the natural cofactor for Cas12a, playing an indispensable role in crRNA pre-assembly, target recognition, and both *cis-* and *trans-*cleavage processes. Its concentration exhibits a direct yet nuanced impact on enzymatic efficiency: while moderately increasing Mg^2+^ can enhance the *tran*s-cleavage activity of Cas12a, excessively high concentrations often promote non-specific cleavage, thereby reducing detection specificity. From a practical perspective, Mg^2+^ is typically optimized within a low-to-moderate millimolar window, where ∼5–10 mM provides a robust baseline for catalytic activity in most *in vitro* detection systems, whereas lower concentrations (≤1 mM) can be selectively employed to enhance mismatch discrimination or regulate specificity at the expense of the reaction rate.

While traditional reaction systems primarily rely on single Mg^2+^ as the sole cofactor, recent studies have revealed that certain alternative divalent metal ions, notably Mn^2+^, can not only substitute for Mg^2+^ but also profoundly enhance Cas12a function by favorably modulating key enzymatic kinetic parameters.^68^ Studies have revealed that Mn^2+^ can markedly accelerate the cleavage kinetics of LbCas12a in systems already containing Mg^2+^. The enhancement mechanism likely stems from the similarities and differences in the chemical properties of Mn^2+^ and Mg^2+^. Although similar in ionic radius, Mn^2+^ possesses partially filled d-orbitals, granting it stronger electrophilicity and greater tolerance for flexible coordination geometries. This property may better stabilize the catalytic transition state, thus improving overall efficiency. Enzymatic kinetic analysis revealed that in the presence of 5 mM Mg^2+^, the addition of Mn^2+^ increased the *trans*-cleavage efficiency (*k*_cat_/*K*_m_) of LbCas12a by approximately 47.6-fold. Specifically, *k*_cat_ increased from 0.035 s^−1^ to 0.546 s^−1^, while *K*_m_ decreased from 2051.45 nM to 662.99 nM, indicating that Mn^2+^ enhances both the catalytic rate and enzyme–substrate affinity. Mn^2+^ also accelerates *cis-*cleavage, reducing the reaction half-life (*τ*_1/2_) by 35%. These results collectively demonstrate that Mn^2+^ comprehensively enhances Cas12a catalysis by optimizing both the catalytic step and the substrate binding step. However, in practical assay design, Mn^2+^ is therefore more suitably used as a low-millimolar supplement to Mg^2+^-containing systems, rather than as a direct universal substitute, so as to achieve a desirable balance between activity enhancement and background suppression.

Similarly, in a MeCas12a (manganese-enhanced Cas12a) system,^151^ Mn^2+^ was found to significantly enhance signal amplification, enabling single-molecule-level detection and accurate discrimination of single-base mutations. This capability improved the differentiation of co-infection cases involving SARS-CoV-2 and MERS-CoV. It is worth noting that the response to such metal enhancement varies among Cas12a orthologs. For instance, Mn^2+^ can induce off-target reporter cleavage in AsCas12a, elevating background even without target presence, whereas LbCas12a remains selective under identical conditions, highlighting the critical importance of ortholog-specific metal ion optimization when employing different Cas12a proteins.

Beyond enhancing activity, metal ions can also modulate Cas12a's tolerance for target sequence mismatches, effectively altering its target recognition specificity. A systematic comparison of the effects of Mg^2+^ on various Cas12a orthologs at physiological concentrations (≤1 mM) found that under low Mg^2+^ conditions,^69^ tolerance for mutations in the PAM and seed regions increased, while tolerance for mutations in the PAM-distal region decreased, exhibiting a specificity “flip”. This metal-dependent switching not only elucidates the discrepancies between *in vivo* and *in vitro* detection but also provides a mechanistic basis for achieving a programmable balance between “targeting and tolerance” through external ion regulation.

Beyond Mg^2+^ and Mn^2+^, other metal ions, including Ca^2+^ and Zn^2+^ can also modulate the activity of the CRISPR/Cas12a system.^152,153^ While their influence is generally less pronounced, evidence confirms their capacity for precise kinetic tuning at optimal concentrations. Unlocking the full potential of these alternative ions represents a promising direction for advancing the performance and specificity of CRISPR/Cas12a systems.^152,154^

### Synergistic effects of organic chemical additives

Small organic molecules can enhance Cas12a activity and expand its target recognition range by fine-tuning the conformation and physicochemical properties of the Cas12a/crRNA/activator complex. Thiol-based reducing agents such as dithiothreitol (DTT) and tri(2-carboxyethyl)phosphine (TCEP), as well as the non-ionic surfactant polyvinyl alcohol (PVA), can increase the distance between key domains within the Cas12a ribonucleoprotein complex by approximately 50%.^155^ These reagents improve enzyme–substrate affinity, reduce protein aggregation, stabilize enzyme conformation, and shorten the detection time by 75–83%, while significantly improving sensitivity. Additionally, DTT substantially relaxes the base-pairing requirements of LbCas12a for the PAM sequence, maintaining high activity output for various non-typical PAMs (*e.g.*, CCTT and AGAT), thereby expanding the targetable sequence space. From an operational standpoint, DTT is typically employed in a low-millimolar regime, with ∼1 mM representing a practical benchmark in commonly used buffer systems, while higher concentrations should be optimized according to assay compatibility and potential redox interference.^70^

### Optimization of phase separation and buffer conditions

Adjusting the macroscopic physicochemical properties of the reaction medium can significantly improve performance without altering protein and nucleic acid components. For instance, adding 15% glycerol to a one-pot RPA-Cas12a system leverages its viscosity to physically isolate the RPA amplification system from the CRISPR components during the early reaction stages.^67^ This prevents Cas12a *cis-*/*trans-*cleavage from prematurely consuming amplification templates or primers, improving sensitivity by nearly two orders of magnitude and achieving aM-level sensitivity comparable to that of a two-step method, while retaining the convenience and low contamination risk of a one-pot format. The successful application of this method provides a new approach for developing highly sensitive, on-site nucleic acid detection platforms.

Furthermore, appropriate buffer systems are essential for maintaining the stability and optimal performance of the CRISPR/Cas12a system. Different buffer components and pH values affect the binding efficiency and reaction kinetics of Cas12a with crRNA, target DNA, and activators. Systematic investigation into reporter probe length and buffer composition revealed that a 15-nt reporter combined with NEB Buffer 4 (containing K^+^ and DTT) increased the reaction rate by approximately 50-fold and lowered the detection limit from nM to pM levels.^71^ This indicates that optimizing the buffer composition can provide a more suitable reaction environment for the CRISPR/Cas12a system, thereby improving its overall detection performance.

In addition to the conditions described above, reaction temperature is another critical factor influencing CRISPR/Cas12a performance. Generally, higher temperatures improve molecular mobility and reaction rates, but excessively high temperatures may lead to protein denaturation or nucleic acid degradation. In practice, the optimal reaction temperature must be determined according to the specific reaction system and detection objectives. For instance, in *in vitro* fluorescence assays, a reaction temperature of around 37 °C often yields the best results, whereas cell-based assays may require temperature adjustments based on cell physiology and experimental requirements to ensure cell viability and detection accuracy.

In summary, precise regulation of external ions and the reaction environment represents an optimization strategy independent of molecular component engineering. It can enhance the catalytic rate, substrate affinity and detection sensitivity of CRISPR/Cas12a without modifying the structures of the crRNA, activator strand, or reporter probe. A key advantage of external environmental regulation is its ability to take effect rapidly without relying on complex molecular modifications and its high orthogonality to strategies such as crRNA optimization, activator strand engineering, and reporter probe design. Metal ions directly alter the reaction energy barrier through coordination at the catalytic center; organic additives optimize the enzyme–substrate spatial relationship *via* conformational rearrangement; and buffer composition and phase separation techniques improve the effective interaction probability by tuning the macroscopic physicochemical properties of the reaction system.

Taken together, these studies demonstrate that the performance evolution of CRISPR/Cas12a biosensing does not rely on a single dominant engineering route, but rather emerges from the coordinated optimization of multiple functional layers, including crRNA, activator strands, reporter probes, and reaction environments. Although each strategy offers distinct advantages in enhancing catalytic activity, suppressing background, improving stability, or enabling exponential signal amplification, their practical utility also differs in terms of mechanistic complexity, ortholog dependence, operational convenience, and application scope. To provide a clearer cross-sectional comparison of the representative approaches discussed in this Review, Table 1 summarizes their engineered components, representative designs, typical applications, key advantages, current limitations, underlying mechanisms, and representative signal-gain performance. This comparison not only helps clarify the distinct functional roles of different molecular engineering strategies, but also highlights their complementary value in constructing next-generation CRISPR/Cas12a diagnostic platforms.

**Table 1:** Comparison of representative engineering strategies for CRISPR/Cas12a-based signal enhancement

Engineered component	Representative design	Typical applications	Key advantages	Current limitations	Primary mechanism	Signal gain (fold-change)
crRNA^78^	3′-end ssDNA extension (7 nt, AT-rich)	SARS-CoV-2 and HIV	Activity enhancement; reduced background; simple modification	Ortholog-dependent efficacy; length and sequence sensitivity	Proximity-driven RuvC catalytic core; autocatalytic cascade	(∼3.5 × ) pM → fM
crRNA^81^	5′-end ssRNA extension (4–9 nt)	HPV16	Improved serum stability; compatible with chemical modifications	AsCas12a-specific optimization required	Conformational change; enhanced RNP stability	aM level
crRNA^89^	Photoactivatable caged crRNA	SARS-CoV-2	Spatiotemporal control; reduced background leakage	Requires external light irradiation	Photocleavage removes the caging group	5–10 × signal to noise
crRNA^74^	Switchable caged crRNA (CONAN)	HBV and bladder cancer mutations	aM sensitivity; single-nucleotide discrimination	Complex circuit design; potential leakage	Autocatalytic positive feedback; target-triggered crRNA liberation	∼363.8 × exponential
crRNA^101^	Split crRNA (asymmetric CRISPR)	miRNA and clenbuterol	Up to 1000-fold amplification; no reverse transcription	Ortholog-dependent splitting tolerance	Competitive binding crRNA; stepwise cascade activation	∼1000×; 856 aM for miRNA
Activator strand^58^	Cir-mediator	DNA and RNA	Dual activator-reporter function; no reverse transcription for RNA	Circular structure synthesis; potential religation	Topological constraint prevents activation; linearization by Cas12a triggers exponential feedback	Exponential
Activator strand^108^	Two stem-loop activators	Pathogen DNA and serum samples	40 min assay time; outperforms qPCR	Cas*Φ* specific; cross-reactivity risk	Structural inhibition; cleavage induced toehold release; cascade activation	0.11 copies per µL (pathogen DNA); 1.2 CFU mL^−1^ in serum
Activator strand^56^	Split activator with LNA modification	BRCA-1 ctDNA	Single base mutation discrimination	LNA synthesis cost; optimization of cleavage sites	LNA-defined cleavage fragments reconstitute activator; positive feedback	4.7 fM
Reporter probe^121^	AIEgen-based reporters	Norovirus and SARS-CoV-2	“One-to-many” signal release	Complex probe synthesis; AIEgen availability	AIEgens intercalated into dsDNA; quencher removal upon cleavage	80–270 × fM to aM levels
Reporter probe^126^	Quencher free 2 AP probe	Goat pox virus, HPV, and ctDNA	“One-to-many” signal release	Moderate quantum yield; sequence-dependent performance	Environment-responsive fluorescence; no external quencher	Not explicitly quantified
Reporter probe^26,132,133^	Metal-enhanced fluorescence with AuNPs	DNA, miRNA, and protein	Visible color change	Precise distance tuning required; nanoparticle synthesis	Plasmonic field enhancement	10 fM (AuNP-SNA); 17.3 fM miRNA; 27.7 pg mL^−1^ PSA
Reporter probe^141^	Hairpin-structured	TdT activity	Exponential output	Requires reverse transcriptase; reaction time longer	Stem-loop conformation enhances substrate affinity	4.55 × 10^−6^ U
Reaction environment^68^	Mn^2+^ supplementation	SARS-CoV-2 and MERS-CoV discrimination	47.6-Fold efficiency increase; 35% *τ*_1/2_ reduction	Ortholog-dependent	Enhanced electrophilicity; better transition-state stabilization	∼47.6 × (*k*_cat_/*K*_m_)
Reaction environment^70,155^	DTT/PVA addition	Non-typical PAM targets	75–83% detection time reduction; relaxed PAM requirement	Additive compatibility; potential interference	Conformational relaxation of the RNP complex; reduced protein aggregation	Not explicitly quantified
Reaction environment^67^	Glycerol phase separation	Nucleic acid POCT	Reduced contamination risk; one-pot convenience	Glycerol concentration optimization	Physical isolation of RPA from Cas12a	∼100 × (*vs.* one-pot without separation)

As the understanding of the CRISPR/Cas12a reaction mechanism deepens, future research will increasingly focus on enhancing the system's reaction speed and detection sensitivity through multi-factor collaborative optimization. For example, combining ion concentration regulation, chemical enhancer addition, and reaction buffer optimization with CRISPR component engineering can construct a more efficient and stable CRISPR/Cas12a biosensor platform. This will provide more convenient and efficient solutions for disease diagnosis, environmental monitoring, and infectious disease control.

## Conclusions and future perspectives

The CRISPR/Cas system represents a revolutionary molecular toolkit that has profoundly transformed the landscape of analytical and diagnostic development. Its unique combination of programmability, specificity, and multifunctionality endows it with unprecedented capabilities in target recognition, cleavage, signal amplification, and *in vivo* imaging. Compared with current diagnostic gold standards such as quantitative PCR (qPCR) and isothermal amplification techniques (*e.g.*, recombinase polymerase amplification and loop-mediated isothermal amplification), which often require thermal cycling, specialized equipment, or exogenous enzymes, CRISPR/Cas12a offers a simpler, isothermal, and more programmable platform with integrated target recognition and signal transduction. More importantly, recent advances in molecular engineering have driven a fundamental shift in the signaling paradigm of Cas12a—from an inherent linear output limited by enzyme turnover to intrinsically amplified systems capable of nonlinear and even exponential signal generation. This transformation establishes CRISPR/Cas12a not merely as a recognition tool, but as a programmable amplification engine, providing a new conceptual framework for achieving ultrasensitive, pre-amplification-free detection in next-generation diagnostic platforms.^156^

In this review, we systematically summarized recent progress in engineering the core components and reaction environment of the CRISPR/Cas12a system to enhance catalytic activity and enable exponential signal amplification. These advances include rational reprogramming of crRNA architecture, activator strand structure, reporter probe design, and external reaction conditions. Collectively, these strategies demonstrate that the analytical performance of Cas12a is not fixed but can be fundamentally reshaped through molecular-level design. In particular, the emergence of autocatalytic circuits, positive feedback loops, multifunctional reporter systems, and structurally reconfigurable activators has enabled Cas12a to evolve from a passive signal transducer into an active amplification module. This transition is of major significance because it opens a feasible route toward amplification-free detection of low-abundance analytes while reducing reliance on conventional upstream nucleic acid amplification procedures.

Despite these advances, several current challenges still hinder the translation of CRISPR/Cas12a-based biosensing systems from laboratory proof-of-concept demonstrations into robust diagnostic technologies suitable for real-world clinical and point-of-care applications. First, matrix interference in complex biological samples (*e.g.*, serum, plasma, and whole blood) remains a major bottleneck. Nonspecific adsorption, nuclease degradation, and background signal leakage can significantly compromise detection accuracy and reproducibility, particularly in highly sensitive amplification systems. Second, the increasing diversity and complexity of molecular engineering strategies introduce system integration and standardization challenges. The coexistence of chemically modified crRNAs, programmable activators, nanomaterial-based reporters, and feedback amplification circuits often leads to compatibility trade-offs, limiting robustness and hindering the establishment of universal design frameworks. Third, large-scale production and manufacturing feasibility remain underexplored. The cost-effective synthesis, purification, and quality control of functional nucleic acids and nanostructured components are essential for clinical deployment but are not yet fully standardized. Fourth, storage stability and operational robustness present additional limitations, as CRISPR components are often sensitive to environmental conditions such as temperature fluctuations and nuclease contamination, restricting their use in point-of-care and resource-limited settings. Last but not least, although remarkable progress has been made in sensitivity enhancement, the development of multiplexed detection systems remains in its infancy. Achieving high-throughput, orthogonal, and interference-resistant multi-target detection requires precise coordination of crRNA specificity, activator orthogonality, and signal readout strategies, representing a significant systems-level challenge.

To address these challenges, future efforts should move beyond isolated component optimization toward holistic system-level engineering. In terms of clinical applicability, the development of anti-interference strategies—such as surface passivation, biomimetic protection, and dynamic background suppression—will be essential for reliable operation in complex biological matrices.^157,158^ Meanwhile, the establishment of standardized and scalable manufacturing protocols, including automated synthesis and rigorous quality control of engineered nucleic acids, will be critical for ensuring reproducibility and facilitating regulatory approval.^159^ Enhancing storage stability through approaches such as lyophilization, encapsulation, or integrated reagent stabilization matrices will further enable long-term storage and field deployment.^160^

In parallel, the realization of multiplexed and high-throughput detection will require the development of orthogonal CRISPR systems, barcoded signal outputs, and advanced signal deconvolution strategies.^161,162^ The integration of CRISPR/Cas12a with microfluidics, paper-based analytical devices, and portable detection platforms is expected to accelerate the development of fully enclosed, automated, and user-friendly point-of-care testing systems. Furthermore, the incorporation of artificial intelligence and machine learning into CRISPR system design and optimization may enable rapid identification of optimal sequences, reaction conditions, and system configurations, thereby significantly accelerating technological iteration.^163^

Overall, the future of CRISPR/Cas12a-based biosensing lies in the seamless integration of molecular innovation with practical application requirements. By bridging the gap between mechanistic understanding and system-level implementation, these technologies are poised to evolve into next-generation diagnostic platforms that are not only ultrasensitive and amplification-free, but also robust, scalable, and widely accessible. Such advances will ultimately enable real-time, multi-target, and intelligent detection across diverse scenarios, marking a significant step forward in the evolution of molecular diagnostics.

## Author contributions

Qing-Nan Li: conceptualization, methodology, investigation, writing – original draft preparation; Qi-Fan Yang: methodology, investigation; Wei-Liang Jin: conceptualization; Xiao-Zhe Pang: investigation; Wen-Bo Sun: investigation; Jia-Xin Wang: investigation; Xin-Yue Wang: investigation; An-Na Tang: methodology, investigation; De-Ming Kong: conceptualization, funding acquisition; Li-Na Zhu: conceptualization, writing – review and editing.

## Conflicts of interest

The authors declare no conflicts of interest.

## Data Availability

No primary research results, software or code have been included and no new data were generated or analysed as part of this review.

## References

[cit1] Kaminski M. M., Abudayyeh O. O., Gootenberg J. S., Zhang F., Collins J. J. (2021). Nat. Biomed. Eng..

[cit2] Gao Y., Gong C., Chen M., Huan S., Zhang X.-B., Ke G. (2024). Anal. Chem..

[cit3] Jia D., Cui M., Ding X. (2024). Small.

[cit4] Zhang Y., Li Q. N., Zhou K., Xu Q., Zhang C. Y. (2020). Anal. Chem..

[cit5] Li Q., Qiang W., Yuan J., Xiao L. (2023). Anal. Chem..

[cit6] Zhao J., Di Z., Li L. (2022). Angew Chem. Int. Ed. Engl..

[cit7] Sheng C., Zhao J., Di Z., Huang Y., Zhao Y., Li L. (2022). Nat. Biomed. Eng..

[cit8] Chen H., Zhou L., Li C., He X., Huang J., Yang X., Shi H., Wang K., Liu J. (2021). Chem. Sci..

[cit9] Grubaugh N. D., Ladner J. T., Lemey P., Pybus O. G., Rambaut A., Holmes E. C., Andersen K. G. (2018). Nat. Microbiol..

[cit10] Zhao Y., Chen F., Li Q., Wang L., Fan C. (2015). Chem. Rev..

[cit11] Vogels C. B. F., Brito A. F., Wyllie A. L., Fauver J. R., Ott I. M., Kalinich C. C., Petrone M. E., Casanovas-Massana A., Catherine Muenker M., Moore A. J., Klein J., Lu P., Lu-Culligan A., Jiang X., Kim D. J., Kudo E., Mao T., Moriyama M., Oh J. E., Park A., Silva J., Song E., Takahashi T., Taura M., Tokuyama M., Venkataraman A., Weizman O.-E., Wong P., Yang Y., Cheemarla N. R., White E. B., Lapidus S., Earnest R., Geng B., Vijayakumar P., Odio C., Fournier J., Bermejo S., Farhadian S., Dela Cruz C. S., Iwasaki A., Ko A. I., Landry M. L., Foxman E. F., Grubaugh N. D. (2020). Nat. Microbiol..

[cit12] Tang Y., Gao L., Feng W., Guo C., Yang Q., Li F., Le X. C. (2021). Chem. Soc. Rev..

[cit13] Paul B., Montoya G. (2020). Biomed. J..

[cit14] Shi Y., Fu X., Yin Y., Peng F., Yin X., Ke G., Zhang X. (2021). Chem.–Asian J..

[cit15] Dai Y., Somoza R. A., Wang L., Welter J. F., Li Y., Caplan A. I., Liu C. C. (2019). Angew. Chem., Int. Ed..

[cit16] Sun W. J., Wang J. Q., Hu Q. Y., Zhou X. W., Khademhosseini A., Gu Z. (2020). Sci. Adv..

[cit17] Zetsche B., Gootenberg J. S., Abudayyeh O. O., Slaymaker I. M., Makarova K. S., Essletzbichler P., Volz S. E., Joung J., vander Oost J., Regev A., Koonin E. V., Zhang F. (2015). Cell.

[cit18] Kirchner M., Schneider S. (2015). Angew. Chem., Int. Ed..

[cit19] Dai Y., Wu Y., Liu G., Gooding J. J. (2020). Angew. Chem., Int. Ed..

[cit20] Yue H., Shu B., Tian T., Xiong E., Huang M., Zhu D., Sun J., Liu Q., Wang S., Li Y., Zhou X. (2021). Nano Lett..

[cit21] Xu Z. C., Chen D. J., Li T., Yan J. Y., Zhu J., He T., Hu R., Li Y., Yang Y. H., Liu M. L. (2022). Nat. Commun..

[cit22] Li S.-Y., Cheng Q.-X., Liu J.-K., Nie X.-Q., Zhao G.-P., Wang J. (2018). Cell Res..

[cit23] Chen J. S., Ma E. B., Harrington L. B., Da Costa M., Tian X. R., Palefsky J. M., Doudna J. A. (2018). Science.

[cit24] Li Q.-N., Wang D.-X., Han G.-M., Liu B., Tang A.-N., Kong D.-M. (2023). Anal. Chem..

[cit25] Li Q.-N., Wang D.-X., Chen D.-Y., Lyu J.-A., Wang Y.-X., Wu S.-L., Jiang H.-X., Kong D.-M. (2024). Anal. Chem..

[cit26] Choi J. H., Lim J., Shin M., Paek S. H., Choi J. W. (2021). Nano Lett..

[cit27] Li Q.-N., Wang D.-X., Dai Z.-Q., Wu S.-L., Han G.-M., Lu S., Zhu L.-N., Jiang H.-X., Kong D.-M. (2024). Chem. Eng. J..

[cit28] Xiong Y., Zhang J., Yang Z., Mou Q., Ma Y., Xiong Y., Lu Y. (2019). J. Am. Chem. Soc..

[cit29] Liang M. D., Li Z. L., Wang W. S., Liu J. K., Liu L. S., Zhu G. L., Karthik L., Wang M., Wang K. F., Wang Z., Yu J., Shuai Y. T., Yu J. M., Zhang L., Yang Z. H., Li C., Zhang Q., Shi T., Zhou L. M., Xie F., Dai H. Q., Liu X. T., Zhang J. Y., Liu G., Zhuo Y., Zhang B. C., Liu C. L., Li S. S., Xia X. K., Tong Y. J., Liu Y. W., Alterovitz G., Tan G. Y., Zhang L. X. (2019). Nat. Commun..

[cit30] Li J., Yang S., Zuo C., Dai L., Guo Y., Xie G. (2020). ACS Sen..

[cit31] Xu S., Wang S., Guo L., Tong Y., Wu L., Huang X. (2023). Anal. Chim. Acta.

[cit32] Wang S. Y., Du Y. C., Wang D. X., Ma J. Y., Tang A. N., Kong D. M. (2021). Anal. Chim. Acta.

[cit33] Li Y., Mansour H., Watson C. J. F., Tang Y., MacNeil A. J., Li F. (2021). Chem. Sci..

[cit34] Zhang Y., Hu C., Yin Y., Ren K., He Y., Gao Y., Han H., Zhu C., Wang W. (2024). Anal. Chem..

[cit35] Zhang Y., Wang W., Zhou X., Lin H., Zhu X., Lou Y., Zheng L. (2024). Anal. Chem..

[cit36] Oesinghaus L., Simmel F. C. (2019). Nat. Commun..

[cit37] Rozners E. (2022). J. Am. Chem. Soc..

[cit38] Fei X., Lei C., Ren W., Liu X., Liu C. (2023). Anal. Chem..

[cit39] Zhao J., Kong D., Zhang G., Zhang S., Wu Y., Dai C., Chen Y., Yang Y., Liu Y., Wei D. (2024). Angew. Chem., Int. Ed..

[cit40] Liu P., Lin Y., Zhuo X., Zeng J., Chen B., Zou Z., Liu G., Xiong E., Yang R. (2024). Angew. Chem., Int. Ed..

[cit41] Huang W., Wang J., Wang C., Liu Y., Li W., Chen Q., Zhai J., Xiang Z., Liu C. (2024). Adv. Sci..

[cit42] Zhao S., Zhang Q., Sun J., Li S., Wang S., Zhou D., Gong X. (2025). Nano Lett..

[cit43] Hu Z., Ling S., Duan J., Yu Z., Che Y., Wang S., Zhang S., Zhang X., Li Z. (2025). Nucleic Acids Res..

[cit44] Zhang D. Y., Winfree E. (2009). J. Am. Chem. Soc..

[cit45] Li H.-D., Fang G.-H., Ye B.-C., Yin B.-C. (2023). Anal. Chem..

[cit46] Ren X. R., Sun W. Q., Li B. W., Xiao Y. J., Yue X. D., Yang M. L., Pang Q. A., Zhu R., Guo Z. Q., Zhang H. P., Wang Y., Liu S., Huang J. D. (2025). Food Control.

[cit47] Cheng L. (2025). Angew. Chem., Int. Ed..

[cit48] Kleinstiver B. P., Sousa A. A., Walton R. T., Tak Y. E., Hsu J. Y., Clement K., Welch M. M., Horng J. E., Malagon-Lopez J., Scarfò I., Maus M. V., Pinello L., Aryee M. J., Joung J. K. (2019). Nat. Biotechnol..

[cit49] Nihongaki Y., Otabe T., Ueda Y., Sato M. (2019). Nat. Chem. Biol..

[cit50] Nguyen L. T., Rananaware S. R., Yang L. G., Macaluso N. C., Ocana-Ortiz J. E., Meister K. S., Pizzano B. L. M., Sandoval L. S. W., Hautamaki R. C., Fang Z. R., Joseph S. M., Shoemaker G. M., Carman D. R., Chang L., Rakestraw N. R., Zachary J. F., Guerra S., Perez A., Jain P. K. (2023). Cell Rep. Med..

[cit51] Li Q., Song Z.-L., Zhang Y., Zhu L., Yang Q., Liu X., Sun X., Chen X., Kong R., Fan G.-C., Luo X. (2023). Anal. Chem..

[cit52] Jiang Y., Qian X., Zheng M., Deng K., Li C. (2023). Microchim. Acta.

[cit53] Hu R., Guo C., Liu C., Zhang Q., Zhang X., Chen Y., Liu Y. (2024). Anal. Chem..

[cit54] Zhang J., Li Z., Guo C., Guan X., Avery L., Banach D., Liu C. (2024). Angew. Chem., Int. Ed..

[cit55] Bagheri N., Chamorro A., Idili A., Porchetta A. (2024). Angew. Chem., Int. Ed..

[cit56] Sun K., Pu L., Chen C., Chen M., Li K., Li X., Li H., Geng J. (2024). Nucleic Acids Res..

[cit57] Hu M., Cheng X., Wu T. (2024). Nucleic Acids Res..

[cit58] Deng F., Li Y., Yang B., Sang R., Deng W., Kansara M., Lin F., Thavaneswaran S., Thomas D. M., Goldys E. M. (2024). Nat. Commun..

[cit59] Yin N., Yu H., Zhang L., Luo F., Wang W., Han X., He Y., Zhang Y., Wu Y., Pu J., Feng T., Yang G., Chen T., Xie G. (2025). Nucleic Acids Res..

[cit60] Ma J.-Y., Wang S.-Y., Du Y.-C., Wang D.-X., Tang A.-N., Wang J., Kong D.-M. (2022). Anal. Chem..

[cit61] Li Q. N., Cui Y. X., Dai Z. Q., Yao Z. L., Li M. Y., Cai Q. L., Kong D. M. (2025). Anal. Chem..

[cit62] Yao Z.-L., Li Q.-N., Li Y.-J., Li H.-R., Yang S.-W., Wang C.-Y., Shi J.-Y., Wu S.-L., Liu S.-Q., Han G.-M., Wang J., Xing L.-Y., Zhao Y., Kong D.-M., Cai Q.-L. (2025). Sens. Actuators, B.

[cit63] Li Q. N., Ma A. X., Wang D. X., Dai Z. Q., Wu S. L., Lu S., Zhu L. N., Jiang H. X., Pang D. W., Kong D. M. (2024). Anal. Chem..

[cit64] Hanson E., Kalla N., Tharu R. J., Demir M. M., Tok B. H., Canbaz M. A., Yigit M. V. (2025). Small.

[cit65] Shi K., Tian Y., Liu S., Luo W., Liu K., Zhang L., Zhang Y., Chang J., Zhang J., Wang S. (2024). Anal. Chim. Acta.

[cit66] Zhao C., Du L., Jiang D., Hu J., Hou X. (2025). Chem. Sci..

[cit67] Lin M., Yue H., Tian T., Xiong E., Zhu D., Jiang Y., Zhou X. (2022). Anal. Chem..

[cit68] Xie S., Xu B., Tang R., Chen S., Lei C., Nie Z. (2022). Anal. Chem..

[cit69] Nguyen G. T., Schelling M. A., Raju A., Buscher K. A., Sritharan A., Sashital D. G. (2024). Nucleic Acids Res..

[cit70] Qing M., Huang C., Li Y., Yu Q., Hu Q., Zhou J., Yuan R., Bai L. (2025). Anal. Chem..

[cit71] Xu J., Liu Z., Zhang Z., Wu T. (2023). Anal. Chem..

[cit72] Dong D., Ren K., Qiu X., Zheng J., Guo M., Guan X., Liu H., Li N., Zhang B., Yang D., Ma C., Wang S., Wu D., Ma Y., Fan S., Wang J., Gao N., Huang Z. (2016). Nature.

[cit73] Swarts D. C., Jinek M. (2019). Mol. Cell.

[cit74] Shi K., Xie S. Y., Tian R. Y., Wang S., Lu Q., Gao D. H., Lei C. Y., Zhu H. Z., Nie Z. (2021). Sci. Adv..

[cit75] Shmakov S. A., Barth Z. K., Makarova K. S., Wolf Y., Brover V., Peters J. E., Koonin E. (2023). Nucleic Acids Res..

[cit76] Shebanova R., Nikitchina N., Shebanov N., Mekler V., Kuznedelov K., Ulashchik E., Vasilev R., Sharko O., Shmanai V., Tarassov I., Severinov K., Entelis N., Mazunin I. (2022). Nucleic Acids Res..

[cit77] Chen Y., Wang X., Zhang J., Jiang Q., Qiao B., He B., Yin W., Qiao J., Liu Y. (2024). Nat. Commun..

[cit78] Nguyen L. T., Smith B. M., Jain P. K. (2020). Nat. Commun..

[cit79] Han J., Park J. S., Kim S., Cha B. S., Lee E. S., Kim J. H., Kim S., Shin J., Jang Y., Chowdhury P., Park K. S. (2022). Microchem. J..

[cit80] Wan Y. Z., Li S., Xu W. F., Wang K., Guo W. L., Yang C. G., Li X. H., Zhou J. H., Wang J. S. (2024). Anal. Chem..

[cit81] Park H. M., Liu H., Wu J., Chong A., Mackley V., Fellmann C., Rao A., Jiang F. G., Chu H. H., Murthy N., Lee K. (2018). Nat. Commun..

[cit82] Ganbaatar U., Liu C. (2022). Sens. Actuators, B.

[cit83] Moon S. B., Kim D. Y., Ko J.-H., Kim J.-S., Kim Y.-S. (2019). Trends Biotechnol..

[cit84] Deleavey G. F., Damha M. J. (2012). Chem. Biol..

[cit85] Eckstein F. (2014). Nucleic Acid Ther..

[cit86] Hendel A., Bak R. O., Clark J. T., Kennedy A. B., Ryan D. E., Roy S., Steinfeld I., Lunstad B. D., Kaiser R. J., Wilkens A. B., Bacchetta R., Tsalenko A., Dellinger D., Bruhn L., Porteus M. H. (2015). Nat. Biotechnol..

[cit87] Ooi K. H., Liu M. M., Tay J. W. D., Teo S. Y., Kaewsapsak P., Jin S., Lee C. K., Hou J., Maurer-Stroh S., Lin W., Yan B., Yan G., Gao Y.-G., Tan M. H. (2021). Nat. Commun..

[cit88] Hu M., Liu R., Qiu Z., Cao F., Tian T., Lu Y., Jiang Y., Zhou X. (2023). Angew. Chem., Int. Ed..

[cit89] Hu M. L., Qiu Z. Q., Bi Z. R., Tian T., Jiang Y. Z., Zhou X. M. (2022). Proc. Natl. Acad. Sci. U.S.A..

[cit90] Ke Y., Huang S., Ghalandari B., Li S., Warden A. R., Dang J., Kang L., Zhang Y., Wang Y., Sun Y., Wang J., Cui D., Zhi X., Ding X. (2021). Adv. Sci..

[cit91] Tan W., Zhang C., Cheng S., Hu X., Wang M., Xian Y. (2024). Anal. Chem..

[cit92] Huang D., He Y., Xu C., Shen P., Li M., Fang M., Xu Z., Fang X. (2025). Anal. Chem..

[cit93] Wu Y., Chang D., Chang Y., Zhang Q., Liu Y., Brennan J. D., Li Y., Liu M. (2023). Small.

[cit94] Li Y., Hu Q., Bai M., Qing M., Bai L. (2025). Anal. Chem..

[cit95] Zhao S., Zhang Q., Luo R., Sun J., Zhu C., Zhou D., Gong X. (2024). Chem. Sci..

[cit96] Xia X., Liang Z., Xu G., Wei F., Yang J., Zhu X., Zhou C., Ye J., Hu Q., Zhao Z., Tang B. Z., Cen Y. (2025). Anal. Chem..

[cit97] Wen M., Zhou M., Huang Z., Wang Y., Wang M., Ding Y., Huang X., Wang B., Wen J., Chen T., Zhang P., Chen M., Yang C., Zhang X.-B., Ke G. (2025). Anal. Chem..

[cit98] Hu H., Guo S., Li Y., Dong K., Lu Y., Ye K., Li L., Zhou X., Cheng L., Xiao X. (2025). Nat. Commun..

[cit99] Zeng J., Liu P., Du J., Li S., Xiong E., Yang R. (2024). Sci. China Chem..

[cit100] Fei X., Lei C., Ren W., Liu C. (2025). Nucleic Acids Res..

[cit101] Moon J., Liu C. (2023). Nat. Commun..

[cit102] Zhang J., Yin W., Jiang Q., Mao W., Deng W., Jin S., Wang X., He R., Qiao J., Liu Y. (2025). Commun. Biol..

[cit103] Liu S., Shi K. (2025). Food Chem..

[cit104] Lei X., Cao S., Liu T., Wu Y., Yu S. (2024). Talanta.

[cit105] Ge H., Wang X., Xu J., Lin H., Zhou H., Hao T., Wu Y., Guo Z. (2021). Anal. Chem..

[cit106] Xia X., Chen Q., Zuo T., Liang Z., Xu G., Wei F., Yang J., Hu Q., Zhao Z., Tang B. Z., Cen Y. (2024). Anal. Chem..

[cit107] Zhou Z., Lau C.-H., Wang J., Guo R., Tong S., Li J., Dong W., Huang Z., Wang T., Huang X., Yu Z., Wei C., Chen G., Xue H., Zhu H. (2024). ACS Omega.

[cit108] Chen H., Song F., Wang B., Huang H., Luo Y., Han X., He H., Lin S., Wan L., Huang Z., Fu Z., Ledesma-Amaro R., Yin D., Mao H., He L., Yang T., Chen Z., Ma Y., Xue E. Y., Wan Y., Mao C. (2025). Nat. Commun..

[cit109] Zhang W., Shi R., Dong K., Hu H., Shu W., Mu Y., Yan B., Li L., Xiao X., Wang H. (2022). Anal. Chem..

[cit110] Zhang Y., Chen Y., Zhang Q., Liu Y., Zhang X. (2023). Biosens. Bioelectron..

[cit111] Snider D. M., Coffin M. L., Armijo B. J., Khetan R., Duchow M. W., Capasso A., Samanta D. (2025). Angew. Chem., Int. Ed..

[cit112] Rananaware S. R., Vesco E. K., Shoemaker G. M., Anekar S. S., Sandoval L. S. W., Meister K. S., Macaluso N. C., Nguyen L. T., Jain P. K. (2023). Nat. Commun..

[cit113] Huang S., Lou Y., Zheng L. (2024). Nucleic Acids Res..

[cit114] Qiao J., Zhang J., Jiang Q., Jin S., He R., Qiao B., Liu Y. (2025). Nucleic Acids Res..

[cit115] Zhou T., Huang R., Huang M., Shen J., Shan Y., Xing D. (2020). Adv. Sci..

[cit116] Zadran S., Standley S., Wong K., Otiniano E., Amighi A., Baudry M. (2012). Appl. Microbiol. Biot..

[cit117] Yuan A. J., Sha R., Xie W. J., Qu G. B., Zhang H. Q., Wang H. L., Le X. C., Jiang G. B., Peng H. Y. (2024). J. Am. Chem. Soc..

[cit118] Yuan A. J., Sun T. R., Chen L. Y., Zhang D. P., Xie W. J., Peng H. Y. (2024). Anal. Chem..

[cit119] Rossetti M., Merlo R., Bagheri N., Moscone D., Valenti A., Saha A., Arantes P. R., Ippodrino R., Ricci F., Treglia I., Delibato E., van der Oost J., Palermo G., Perugino G., Porchetta A. (2022). Nucleic Acids Res..

[cit120] Tang X., Zhu Y., Guan W., Lu C. (2023). Aggregate.

[cit121] Guo Y., Zhou Y., Duan H., Xu D., Wei M., Wu Y., Xiong Y., Chen X., Wang S., Liu D., Huang X., Xin H., Xiong Y., Tang B. Z. (2024). Nat. Commun..

[cit122] Gootenberg J. S., Abudayyeh O. O., Lee J. W., Essletzbichler P., Dy A. J., Joung J., Verdine V., Donghia N., Daringer N. M., Freije C. A., Myhrvold C., Bhattacharyya R. P., Livny J., Regev A., Koonin E. V., Hung D. T., Sabeti P. C., Collins J. J., Zhang F. (2017). Science.

[cit123] Gootenberg J. S., Abudayyeh O. O., Kellner M. J., Joung J., Collins J. J., Zhang F. (2018). Science.

[cit124] Broughton J. P., Deng X. D., Yu G. X., Fasching C. L., Servellita V., Singh J., Miao X., Streithorst J. A., Granados A., Sotomayor-Gonzalez A., Zorn K., Gopez A., Hsu E., Gu W., Miller S., Pan C. Y., Guevara H., Wadford D. A., Chen J. S., Chiu C. Y. (2020). Nat. Biotechnol..

[cit125] Li S. Y., Cheng Q. X., Wang J. M., Li X. Y., Zhang Z. L., Gao S., Cao R. B., Zhao G. P., Wang J. (2018). Cell Discov..

[cit126] Chen X., Huang C., Hu Q., Zhang J., Wang D., You Q., Hu M. (2023). Analyst.

[cit127] Uhlmann E., Ryte A., Peyman A. (1997). Antisense Nucleic Acid Drug Dev..

[cit128] Cutler J. I., Auyeung E., Mirkin C. A. (2012). J. Am. Chem. Soc..

[cit129] Seferos D. S., Prigodich A. E., Giljohann D. A., Patel P. C., Mirkin C. A. (2009). Nano Lett..

[cit130] Shi L., Jing C., Ma W., Li D. W., Halls J. E., Marken F., Long Y. T. (2013). Angew. Chem., Int. Ed..

[cit131] Hwang J.-H., Park S., Son J., Park J. W., Nam J.-M. (2021). Nano Lett..

[cit132] Wang C., Xu X., Yao W., Wang L., Pang X., Xu S., Luo X. (2025). Nano Lett..

[cit133] Fu X., Shi Y., Peng F., Zhou M., Yin Y., Tan Y., Chen M., Yin X., Ke G., Zhang X.-B. (2021). Anal. Chem..

[cit134] Zhou M., Yin Y., Shi Y., Huang Z., Shi Y., Chen M., Ke G., Zhang X.-B. (2022). Chem. Commun..

[cit135] Freko S., Nikić M., Mayer D., Weiß L. J. K., Simmel F. C., Wolfrum B. (2024). ACS Sen..

[cit136] Lee Y., Choi J., Han H. K., Park S., Park S. Y., Park C., Baek C., Lee T., Min J. (2021). Sens. Actuators, B.

[cit137] Zhang D. C., Yan Y. R., Que H. Y., Yang T. T., Cheng X.
X., Ding S. J., Zhang X. M., Cheng W. (2020). ACS Sen..

[cit138] Li F., Li J., Yang W., Yang S., Chen C., Du L., Mei J., Tang Q., Chen X., Yao C., Yang D., Zuo X., Liu P. (2023). Angew. Chem., Int. Ed..

[cit139] Mocenigo M., Porchetta A., Rossetti M., Brass E., Tonini L., Puzzi L., Tagliabue E., Triulzi T., Marini B., Ricci F., Ippodrino R. (2020). ACS Sen..

[cit140] Vallee-Belisle A., Ricci F., Plaxco K. W. (2009). Proc. Natl. Acad. Sci. U. S. A..

[cit141] Meng T., Kang Q., Xu J., Zhao S., Liu T., Zhou D., Gong X., Zhang J. (2025). Talanta.

[cit142] Lee S., Nam D., Park J. S., Kim S., Lee E. S., Cha B. S., Park K. S. (2022). Biochip. J..

[cit143] Tian Y., Chen J. X., Chen F. Z., Xu J. R., Huang L. X., Peng L. I., Li H. L., Shi K. (2025). Anal. Chim. Acta.

[cit144] Wu Y., Yue Y., Deng S., He G., Gao H., Zhou M., Zhong K., Deng R. (2020). J. Agric. Food Chem..

[cit145] Xia X., Ma B., Zhang T., Lu Y., Khan M. R., Hu Y., Lei C., Deng S., He Q., He G., Zhang K., Deng R. (2021). ACS Sens..

[cit146] Li T., Hu R., Xia J., Xu Z., Chen D., Xi J., Liu B.-F., Zhu J., Li Y., Yang Y., Liu M. (2021). Biosens. Bioelectron..

[cit147] Chen Y., Ma X., Pan L., Yang S., Chen X., Wang F., Yang D., Li M., Wang P. (2024). Sens. Actuators, B.

[cit148] Jinek M., Jiang F., Taylor D. W., Sternberg S. H., Kaya E., Ma E., Anders C., Hauer M., Zhou K., Lin S., Kaplan M., Iavarone A. T., Charpentier E., Nogales E., Doudna J. A. (2014). Science.

[cit149] Huang X., Sun W., Cheng Z., Chen M., Li X., Wang J., Sheng G., Gong W., Wang Y. (2020). Nat. Commun..

[cit150] Hood M. I., Skaar E. P. (2012). Nat. Rev. Microbiol..

[cit151] Ma P., Meng Q., Sun B., Zhao B., Dang L., Zhong M., Liu S., Xu H., Mei H., Liu J., Chi T., Yang G., Liu M., Huang X., Wang X. (2020). Adv. Sci..

[cit152] Son H., Park J., Choi Y. H., Jung Y., Lee J. W., Bae S., Lee S. (2022). Chem. Commun..

[cit153] Li B., Yan J. Y., Zhang Y. X., Li W. Q., Zeng C. X., Zhao W. Y., Hou X. C., Zhang C. X., Dong Y. Z. (2020). Mol. Ther. Nucl. Acids.

[cit154] Wu S., Liu Y. C., Zeng T. Y., Zhou T. C., Sun Y. T., Deng Y., Zhang J., Li G. X., Yin Y. M. (2025). Adv. Sci..

[cit155] Deng F., Li Y., Li B., Goldys E. M. (2022). Sens. Actuators, B.

[cit156] Ghouneimy A., Mahas A., Marsic T., Aman R., Mahfouz M. (2023). Acs Synth. Biol..

[cit157] Yu Y., Zhang Y., Zhao Y. N., Lv K. Z., Ai L. Z., Wu Z. J., Song Z. B., Zhang J. (2025). Biosens. Bioelectron..

[cit158] Li C. Y., Zheng B., Lu L. L., Fang W. K., Zheng M. Q., Gao J. L., Liu Y. H., Pang D. W., Tang H. W. (2021). Anal. Chem..

[cit159] Goméz-Quintero O. S., Morales-Moreno M. D., Valdés-Galindo E. G., Cárdenas-Guerra R. E., Hernandez-Garcia A. (2025). Protein Expres. Purif..

[cit160] Wang Y., Xu X. N., Que J. Q., Wang X. Y., Ni W., Wu Y. H., Yang L., Li Y. (2024). Anal. Chem..

[cit161] Jia H. Y., Zhang X. Y., Ye B. C., Yin B. C. (2024). Anal. Chem..

[cit162] Li S. Q., Jin B. C., Ma Y. T., Yang X., Fan J. L., Xie Y. L., Xu C. L., Dai X., Wang M. J., Liu Q. Q., Fu T., Liu Y., Tan W. H. (2024). J. Am. Chem. Soc..

[cit163] Li Q., Lin X. D. (2026). TrAC, Trends Anal. Chem..

